# Influence of *Bacillus subtilis* strain Z-14 on microbial communities of wheat rhizospheric soil infested with *Gaeumannomyces graminis* var. *tritici*

**DOI:** 10.3389/fmicb.2022.923242

**Published:** 2022-09-02

**Authors:** Zhaosha Liu, Jiawen Xiao, Xuechao Zhang, Shijuan Dou, Tongguo Gao, Dongmei Wang, Dongdong Zhang

**Affiliations:** ^1^College of Life Science, Hebei Agricultural University, Baoding, China; ^2^Hebei Provincial Engineering Research Center for Resource Utilization of Agricultural Wastes, Baoding, China; ^3^State Key Laboratory of North China Crop Improvement and Regulation, Baoding, China

**Keywords:** *Bacillus subtilis*, *Gaeumannomyces graminis* var. *tritici*, microbial carbon metabolism, soil microbial diversity, whole-genome sequencing

## Abstract

Wheat take-all disease caused by *Gaeumannomyces graminis* var. *tritici* (*Ggt*) spreads rapidly and is highly destructive, causing severe reductions in wheat yield. *Bacillus subtilis* strain Z-14 that significantly controlled wheat take-all disease effectively colonized the roots of wheat seedlings. Z-14 increased the metabolic activity and carbon source utilization of rhizospheric microorganisms, thus elevating average well-color development (AWCD) values and functional diversity indexes of soil microbial communities. Z-14 increased the abundance of *Bacillus* in the rhizosphere, which was positively correlated with AWCD and functional diversity indexes. The Z-14-treated samples acquired more linkages and relative connections between bacterial communities according to co-occurrence network analyses. After the application of *Ggt*, the number of linkages between fungal communities increased but later decreased, whereas Z-14 increased such interactions. Whole-genome sequencing uncovered 113 functional genes related to Z-14’s colonization ability and 10 secondary metabolite gene clusters in the strain, of which nine substances have antimicrobial activity. This study clarifies how bacterial agents like Z-14 act against phytopathogenic fungi and lays a foundation for the effective application of biocontrol agents.

## Introduction

Wheat take-all, a soil-borne plant fungal disease, caused by *Gaeumannomyces graminis* var. *tritici* (*Ggt*; Freeman and Ward, 2004), is an important root disease in major wheat-producing areas worldwide (Keenan et al., 2015)*. Ggt* also infects corn, oat, barley, triticale, and other gramineous crop plants, adversely affecting their yield (Bithell et al., 2011). Biological control impacts the environment little, does not promote crop drug resistance, and is relatively safe for people and animals, which aligns well with sustainable agricultural development and broad application prospects for wheat take-all prevention (Brian and David, 2001; Rao et al., 2016; Thakur et al., 2022).

*Bacillus* species, widely abundant in plants and natural environments, can effectively inhibit a variety of pathogenic fungal diseases which infect roots, leaves, flowers, and even fruits (Nam et al., 2016). The endospores formed by the *Bacillus* genus are strongly heat resistant and tolerant to desiccation. These properties have enabled their development as easily stored commercial products with a long shelf life (Posada et al., 2016). *Bacillus* spp. produce an array of antimicrobial substances conducive to the colonization of plants and rhizospheric soil and functioning as biocontrol agents, making them ideal microorganisms for controlling plant diseases (Ongena and Jacques, 2008; Wang et al., 2014). A known plant growth promoter, *B. amyloliquefaciens* FZB42 applied to roots of greenhouse- or indoor-cultivated seedlings, shrubs, and decorative plants can control root rot and blight caused by *Fusarium* and *Rhizoctonia* (Chen et al., 2007). Recently, Sun et al. (2019) screened *B. amyloliquefaciens* SW-34 from rhizospheric soil of ginseng and conducted greenhouse pot experiments on its control of ginseng gray mold; they found disease resistance post-inoculation with SW-34 reached 75%, this exceeding the control effect of chemical fungicides.

Soil microorganisms underpin soil biological fertility, which not only regulates the development of plants and inhibits the growth of phytodisease-causing microorganisms but also promotes the cycling of nutrient elements required by plants, the maintenance of soil fertility, and energy transformations (Li et al., 2017). Achieving a dynamic equilibrium between plant rhizospheric soil microorganisms and plants is of great significance for plants’ disease resistance and growth, since pathogen invasions can directly induce diseases and weaken them against other enemies (Chen et al., 2020). Biocontrol microorganisms are used to supplement the microbiota, to establish a new micro-ecological equilibrium state, by inhibiting the activity of plant pathogens and preventing disease outbreaks (Podolich et al., 2015). For example, a *Trichoderma* agent inhibits *Phytophthora* infection of pepper seeds by changing the microbiota structure in the rhizosphere (Ros et al., 2017); *B. amyloliquefaciens* B1408 is able to augment cucumber’s growth and lessen its damage from Fusarium wilt by changing the composition of its rhizospheric microbiota (Han et al., 2019).

In recent years, many studies have applied *Bacillus* spp. to control phytopathogenic fungi and reported many positive achievements. However, application strategies for biocontrol agents to improve the functional and structural diversity of soil microorganisms conducive to plant growth still lack a systematic practice and theoretical basis. Accordingly, this study aimed to comprehensively explore the influence of *B. subtilis* strain Z-14 and its application strategy on the functional and structure diversities of rhizospheric soil. The goal was to make better use of biocontrol agents for preventing plant diseases.

## Materials and methods

### Strains

The biocontrol bacterium *B. subtilis* Z-14, which can inhibit the proliferation of many pathogenic fungi and is capable of controlling wheat take-all disease caused by *Ggt*. Strain Z-14 was preserved in the Pharmaceutical Engineering Laboratory of Hebei Agricultural University (Zhang et al., 2017b; Xiao et al., 2021). A single colony of strain Z-14 activated in nutrient agar overnight was inoculated in nutrient broth (NB) and incubated overnight at 37°C and 180 rpm, then transferred into fresh NB medium with a 10% inoculation amount and cultured at 37°C and 180 rpm for 48 h. This cultured Z-14 broth was then diluted with sterile water to 1.0 × 10^8^ colony forming units (cfu)/ml for wheat seeds’ soaking and soil irrigation. The preparation of fungal pathogen *Ggt* inoculum and its subsequent inoculation to soil followed the protocol described by Zhang et al. (2017a).

### Pot experiment design

A 2.0-g sample of ground *Ggt*-colonized oat kernels samples was placed onto the surface of non-sterile soil (500 g) pre-loaded into a plastic pot (13-cm diameter, 12.5-cm height), and covered with a layer (0.5 cm) of non-sterile soil (pH 7.83, organic matter 19.97 g/kg, total *N* 2.31 g/kg, available P 49.18 mg/kg, available K 201.35 mg/kg, cation exchange capacity 16.27 cmol/kg, sand 12.3%, silt 78.5%, and clay 9.2%). Wheat seeds (cultivar ‘shi4185’) previously soaked with the Z-14 broth for 2 h. Approximately 1.0 × 10^5^ cfu per seed of strain Z-14 were detected using the standard plate counting method. Ten pre-germinated seeds were sown in each pot and covered with a layer (1 cm) of non-sterile soil collected from the experimental farm of Hebei Agricultural University’ west campus (Baoding, China). Next, 20 ml of Z-14 broth was uniformly sprayed onto the soil, and then another thin layer (1 cm) of non-sterile soil was sprinkled on top to cover the bacterial agent. Four treatments were set up in the experiment: CK0 (non-microorganism-inoculated), CK1 (only *Ggt*-inoculated), and Z1 and Z2 (inoculated with both *Ggt* and Z-14). Seven days after sowing, every pot in the Z2 treatment was irrigated with 20 ml of Z-14 broth. Each treatment had 15 replicate pots arranged in a randomized complete block design and cultured in a growth chamber in 16-h/8-h light/darkness at 24°C. After 4 weeks of growth, the disease index (DI) and disease reduction (DR) of wheat take-all were calculated, as described in Zhang et al. (2017a). Based on the percentage of root area affected by *Ggt*, disease severity was assigned to one of five classes: class 0 = 0%, class 1 = 1–10%, class 2 = 11–30%, class 3 = 31–60%, and class 4 = 61–100%.


$$DI=\overset{4}{\underset{i=0}{\sum }}\left(n_{i}\times i\right)\times \overset{4}{\underset{i=0}{\sum }}{\left(n_{i}\right)}^{-1}$$

in which *i* = severity class and *n_i_* = number of plants assigned to the class *i*. The DI values calculated ranged from 0 (no disease) to 100 (highest disease level). Disease reduction (DR) was calculated (%) as 100 × (DI-*Ggt*−DI-test)/DI-*Ggt*. Fresh weights and root and shoot lengths were also determined.

### Transmission electron microscopy observations

Harvested wheat roots from each treatment were cut into small 2-mm-long segments, which were immersed in 2.5% glutaraldehyde in 0.1 mol/l phosphate-buffered saline (PBS, pH 7.4) for 24 h at 4°C. The samples were removed from PBS and rinsed with fresh PBS. They were then fixed in 1% osmium tetroxide solution for 1 h at 37°C. The postfixed samples were dehydrated with increasing concentrations of ethyl alcohol (75, 85, 95, and 100%), infiltrated with a propylene oxide-Araldite mixture, and embedded in Araldite. The segments were sectioned using a Leica EM UC7 ultramicrotome. Ultrathin sections of tissue (50 nm) were mounted onto copper grids and stained with uranyl acetate and lead citrate for 20 min each. Ultrastructure variation in wheat roots was observed to evaluate strain Z-14’s colonization ability and control effect against wheat take-all by using a H-7650 TEM (Hitachi; Xiao et al., 2021; Bhatia et al., 2022).

### Effects of *Bacillus subtilis* Z-14 on soil microbial functional diversity

At 9 and 19 days, after seeds were sown, rhizospheric soil from each treatment’s pots was collected separately, to assess its microbial functional and structural diversity. The metabolic functional diversity of soil microbial communities was analyzed using Biolog Eco-plates with 31 different organic substrates (Supplementary Table S1; Ge et al., 2018). For this, a fresh soil sample (1.0 g) was added into 9 ml of a 0.85% stroke-physiological saline solution. This mixture was oscillated and shaken at 200 rpm and 4°C for 30 min, then allowed to rest for 5 min. Each bacterial suspension was first diluted to a 10^−3^ gradient and then added (150 μl) to the Biolog Eco-Plate (Biolog, Hayward, United States). Microplates were cultured at 28°C for 10 days, with their absorbance at 590 and 750 nm read every 24 h by a customized microplate reader (ELx808^™^, Biolog, United States). The capability of microorganisms to utilize different carbon sources in the microbial communities was measured by average well-color development (AWCD), this was calculated as the mean of absorbance values for 31 carbon sources. Functional diversity of soil bacterial communities was expressed by the Pielou evenness (E), Shannon-Wiener diversity (H′), Simpson dominance (D), and McIntosh diversity (U) indexes.

### High-throughput sequencing of soil samples’ DNA and data processing

Microbial community genomic DNA was extracted from soil samples with the E.Z.N.A.^®^ soil DNA Kit (Omega Bio-Tek, United States), according to the manufacturer’s instructions. 16S rRNA gene sequencing was used for bacterial profiling, in which the bacterial 16S gene V3 ~ V4 region was PCR-amplified by using the primers 338F and 806R. The modified primers ITS1F and ITS2 were used to amplify the first fungal internal transcribed spacer (ITS1) region (Supplementary Table S2). The 16S and ITS1 amplicons were sequenced at the Majorbio Bio-pharm Technology Co., Ltd. (Shanghai, China) on the Illumina MiSeq PE300 sequencing system (Illumina, United States; Qiao et al., 2018). Raw gene sequencing reads were demultiplexed, quality-filtered by fastp v0.20.0 (Chen et al., 2018), and merged by FLASH v1.2.7 (Magoc and Salzberg, 2011). Operational taxonomic units (OTUs) with a 97%-similarity cutoff were clustered using UPARSE v7.1 (Edgar, 2013), and chimeric sequences were identified and discarded. Each OTU representative sequence was taxonomically analyzed by RDP Classifier v2.2 (Wang et al., 2007) against the 16S rRNA and ITS1 databases (e.g., Silva v138), using a confidence threshold of 0.7 (Chen et al., 2019). The raw dataset of the sequencing result has been submitted to the NCBI BioProject repository.^1^

### Genome sequencing and functional genomic analysis of *Bacillus subtilis* Z-14

A single colony of strain Z-14 was inoculated in NB medium and cultured overnight (37°C, 180 rpm). The supernatant was removed by centrifugation at 8000 × *g* for 10 min, and the bacterial genome was extracted using a Bacterial Genome DNA Extraction Kit (Tiangen Biotech, China). Genomic DNA was sequenced using a combination of PacBio RS II Single Molecule Real Time (SMRT) and Illumina sequencing platforms. The original image data were transferred into sequence data *via* base calling, which was defined as raw reads, and saved as an FASTQ file. A quality information statistic was applied for quality trimming, by which the low-quality data can be removed to form clean data. The reads were then assembled into a contig using hierarchical genome assembly process (HGAP) and canu. The last circular step was checked and finished manually, generating a complete genome with seamless chromosomes and plasmids. Finally, error correction of the PacBio assembly results was performed with Pilon using the Illumina reads. Glimmer was used for CDS prediction, tRNA-scan-SE was used for tRNA prediction, and Barrnap was used for rRNA prediction. The predicted CDSs were annotated from three databases: Gene Ontology (GO; Ashburner et al., 2000), Kyoto Encyclopedia of Genes and Genomes (KEGG; Kanehisa et al., 2006), and Cluster of Orthologous population Groups of proteins (COG; Tatusov et al., 2003), to functional annotate the genes using BLAST software. According to the KEGG classification of metabolic pathways, those genes related to rhizospheric activities of *B. subtilis*, such as colonization, growth promotion, and induced systemic resistance in the genome of strain Z-14 were identified to preliminarily evaluate its biocontrol potential. Secondary metabolic gene clusters, their sequence characteristics, and their existence in microbial genomes were analyzed using AntiSMASH3.0 (Medema et al., 2011). The raw dataset of the sequencing result has been submitted to the NCBI BioProject repository.^2^

### Statistical analysis

Data were analyzed on the Majorbio Cloud Platform.^3^ The optimized data of the samples were analyzed at each taxonomic level, for which a community composition histogram was obtained. Mothur v.1.30.2 and its summary single command were used to calculate the alpha diversity indexes. Circos diagrams displaying the relationship between samples and species were generated in Circos-0.67-7.^4^ Principal component analysis (PCA) was employed to evaluate the differences of carbon source utilization and relative abundances of fungal and bacterial genera, respectively. PCA was conducted with vegan package in the R computing platform (v3.3.1) based on the Euclidean distance metric. Spearman rank correlations between the Bray–Curtis dissimilarity measures of soil microbial functional diversity and structural diversity were performed in a heatmap with the “pheatmap” package (version 1.0.12) in R (version 4.0.4). A valid co-occurrence was selected as a strong correlation if the Spearman’s correlation coefficient (ρ) calculated with the “psych” package (version 2.0.12) in R was >0.6 with a corrected significance level < 0.01. Correlation networks between genera in the samples were constructed and visualized using Cytoscape software (v 3.2.1). Data from replicates were expressed as means ± standard deviation (SD). The SPSS v17.0 software (IBM Corp., United States) was used to perform the calculations and compare the means of the four experimental treatments. Significant differences between them were assessed using Duncan’s HSD test and one-way analysis of variance (ANOVA; significant at *p*-value <0.05).

## Results and discussion

### Biocontrol effect of *Bacillus subtilis* Z-14 against wheat take-all

**Figure 1:**
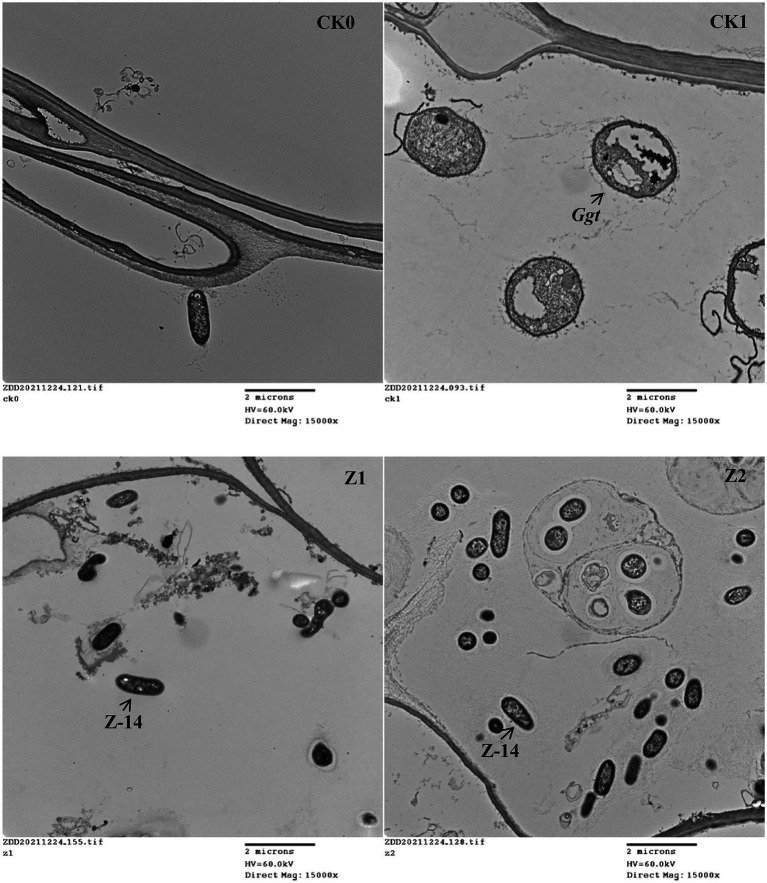
The TEM (transmission electron microscope) observations of root tissues from wheat seedlings inoculated with the fungus *Gaeumannomyces graminis* var. *tritici* and *Bacillus subtilis* strain Z-14 after wheat seeds sown for 4 weeks. **A** represented CK0, no microorganism inoculated; **B** represented CK1, only *Ggt* inoculated; **C** represented Z1, inoculated with *Ggt* and Z-14 when seeds were sown; **D** represented Z2, treated the same as Z1 and each pot was irrigated with 20 ml of Z-14 broth on the 7th day after sowing.

The control efficacy of strain Z-14 against wheat take-all reached 52.56% under treatment Z1 and 66.25% under treatment Z2 (Table 1). Wheat seedlings treated with Z-14 had a significantly greater root length and shoot height, as well as root and shoot fresh weights than did CK1 seedlings inoculated with *Ggt*. Compared with Z1, the Z2 treatment significantly increased the root fresh weight; root length and root and shoot weights also increased, but these differences were not significant. TEM revealed that, compared with CK0 (Figure 1A), many fungal hyphae of *Ggt* were detected in the seedling roots of CK1 (Figure 1B). The observation indicated that *Ggt* hyphae infect the root of wheat seedlings. Detecting seedling roots of Z1 revealed that the infection was inhibited by Z-14. The fungal hyphae were reduced and some Z-14 bacteria were observed (Figure 1C). More Z-14 bacteria and fewer fungal hyphae of *Ggt* were observed in seedling roots of Z2 compared with those of Z1 (Figure 1D). These findings demonstrated effective colonization in the roots of wheat seedlings and significant inhibition of *Ggt* hyphae by Z-14 in a manner that was dependent on bacterial inoculation frequency.

**Table 1:** Control effect of *Bacillus subtilis* strain Z-14 on wheat take-all disease of wheat seedlings caused by the *G. graminis* var. *tritici* fungus.

Tr	RL (cm)	RFW (mg)	AH (cm)	AFW (g)	DI (%)	DR (%)
CK0	16.63 ± 0.80a	121.16 ± 7.89a	27.62 ± 0.98a	284.15 ± 14.36a	–	–
CK1	9.27 ± 1.75c	69.24 ± 12.93c	19.24 ± 0.85c	162.21 ± 20.68c	63.59 ± 7.63a	–
Z1	14.25 ± 0.68b	92.56 ± 9.98b	22.23 ± 1.61b	234.63 ± 21.49b	30.17 ± 4.56b	52.56
Z2	15.81 ± 1.92ab	112.65 ± 7.12a	25.15 ± 2.28ab	265.72 ± 31.40ab	21.46 ± 5.43c	66.25

CK0, no microorganism inoculated; CK1, only Ggt inoculated; Z1, inoculated with Ggt and Z-14 when wheat was planted; Z2, treated the same as Z1 and each pot was irrigated with 20 ml of Z-14 broth on the 7th day after sowing. Tr, Treatment; RL, root length; RFW, root fresh weight; AH, above height; AFW, above fresh weight; DI, disease index; DR, disease reduction; Different lowercase letters within the same column indicate significant differences between treatments at the 5% level (*p* < 0.05).

*Bacillus subtilis* plays a prominent role in preventing plant diseases in various ways, through competition, antagonism, inducing plant resistance, or promoting plant growth (Allard-Massicotte et al., 2016; Hoffmann et al., 2021). In any case, maintaining enough biocontrol bacteria in the rhizosphere is needed for them to exert significant control effects (Townsley et al., 2018). Foreign bacteria could be maintained at high levels in soil through their continuous addition, to ensure the sought-after biocontrol effect (Han et al., 2021). In a field experiment, vascular symptoms and the relative abundance of *Fusarium oxysporum* in the root endosphere of *F. oxysporum* f. sp. *lycopersici* (FOL)-challenged plants were reduced by adding, twice, a mixture of *Collimonas arenae* Cal35 and Serenade Soil (Hung et al., 2020). Here, by adding the biocontrol agent Z-14 to soil, its effect for controlling wheat take-all was improved. Meanwhile, the TEM results indicated the number of bacteria in wheat root increased with the application of strain Z-14, consistent with its greater control effect against wheat take-all. These results suggested the biocontrol strain’s abundance in wheat roots was increased by continuous addition, which strengthened its control effect. However, a continuous addition strategy increases planting costs, and the feasibility of practical application depends on the increased yield in field production.

### Effects of *Bacillus subtilis* Z-14 on soil diversity of microbial community function

Carbon source utilization by microbes in wheat rhizospheric soil under the different treatments was investigated (Supplementary Figure S1). Soil samples from different phases showed similar detection results, in that Z2 and CK0 attained the maximum and minimum value, respectively, while the AWCD value of Z1 surpassed that of Z2. On day 9 after seeds were sown, significant differences of AWCD values were observed between CK0 and the other treatments. No significant difference was observed among CK1, Z1, and Z2. On day 19 after seeds were sown, significant differences of AWCD values between Z2 and the other treatments (except Z1) were evident, yet CK0, CK1, and Z1 had similar values. Likewise, carbon source utilization by rhizomicrobes of cucumber after fungus *Trichoderma longibrachiatum* T2 was applied to soil was enhanced at the seedling stage (Li et al., 2013). Adding Z-14 significantly increased the metabolic activity and carbon source utilization of soil microorganisms, resulting in higher AWCD values of Z1 and Z2 compared with CK0 and CK1. Compared with the sampling on the 9th day, the AWCD value on the 19th day was significantly higher under Z2 than the other treatments except Z1; hence, more Z-14 bacteria were retained in soil after multiple inoculations, this is consistent with the strain’s biocontrol effect and TEM observations.

**Figure 2:**
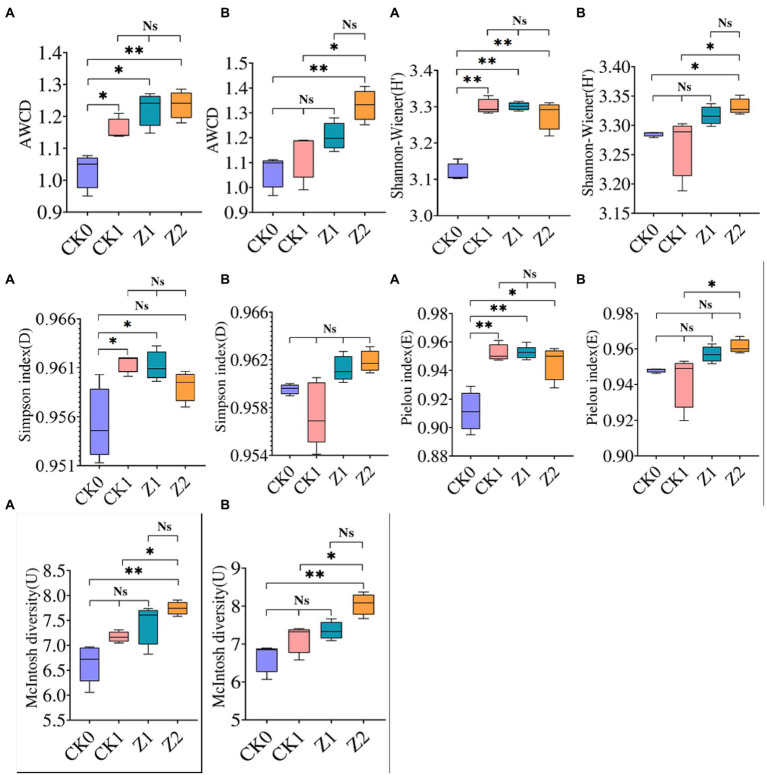
AWCD and functional diversity indexes of microbial communities from wheat rhizospheric soil sampled on the 9th day **(A)** and 19th day **(B)** after sowing when incubated for 216 h. CK0, no microorganism inoculated; CK1, only *Ggt* inoculated; Z1, inoculated with *Ggt* and Z-14 when seeds were sown; Z2, treated the same as Z1 and each pot was irrigated with 20 ml of Z-14 broth on day 7 after sowing. Ns, not significant, ^*^p < 0.05, ^**^p < 0.01.

For microbial communities of soil samples collected on the 9th day after seeds were sown, the H′, D (except Z2), and E indices of CK0 after inoculation for 216 h were significantly lower than the other treatments, the latter being similar (Figure 2). The application of pathogens and antagonists significantly increased the diversity and evenness of soil microorganisms, and the ecological functioning of dominant organisms was more pronounced. For microbial communities of soil sampled on the 19th day after inoculation for 216 h, their H′ and E indices were highest under Z2 and lowest under CK1. The differences between the two treatments were significant. The Z2 treatment had the largest U index, significantly different from CK0 and CK1, whereas the U index was similar for Z1 vis-à-vis the other treatments. Among the four treatments, all four indexes were greatest under Z2 at 19 days post-sowing, indicating that adding Z-14 increased the multidimensional spatial uniformity, diversity of carbon source utilization, dominance of communities, and species evenness of soil microbial communities. In other research, community-level physiological profiling of yeast *Cryptococcus* sp. NSE1 with plant growth-promoting capabilities found it increased the H′, D, and U of the microbial community (Liu et al., 2016). On the contrary, the influence of 1-octyl-3-methylimidazolium hexafluorophosphate on the soil microbial community diversity demonstrated that AWCD and the diversity indexes were significantly reduced (Zhang et al., 2018). In sum, substances beneficial to soil microbial communities promote microbial diversity, while harmful substances diminish it.

**Figure 3:**
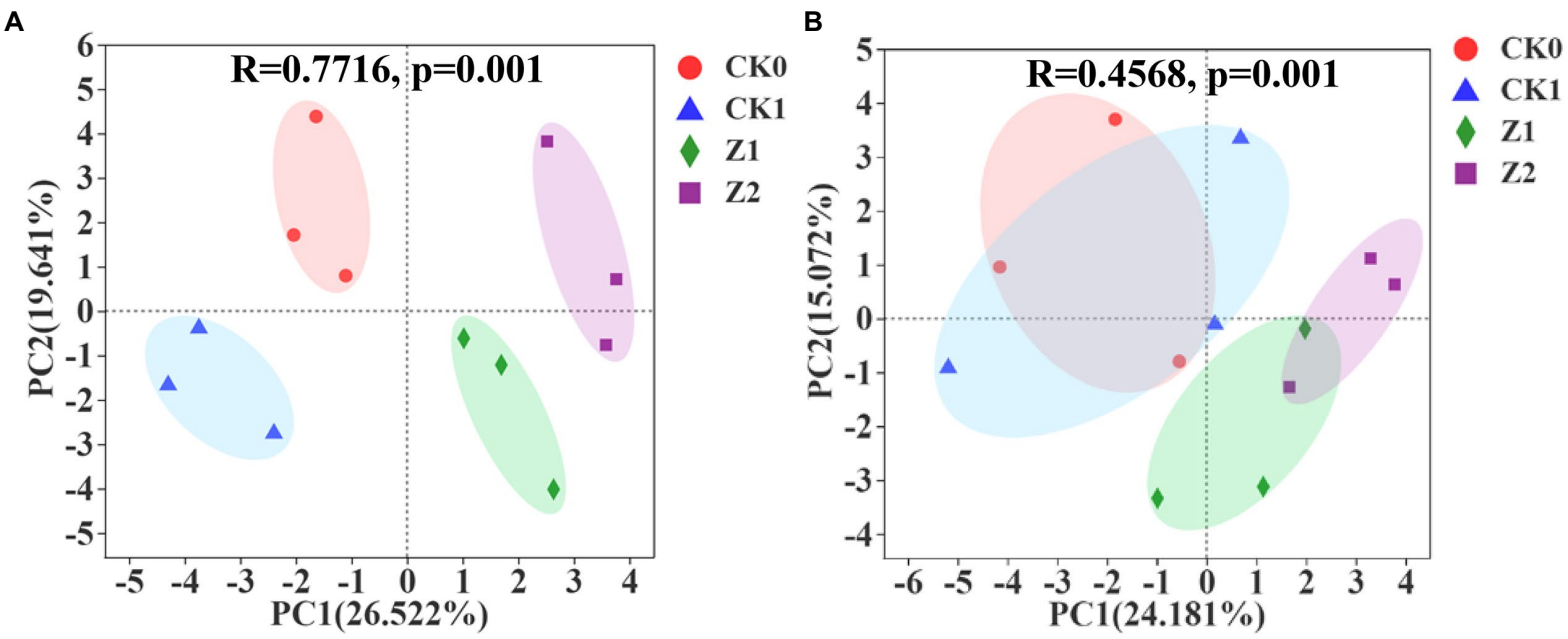
Principal component analysis of microbial metabolic function of the wheat rhizosphere sampled on the 9th **(A)** and 19th day **(B)** after sowing when incubated for 216 h. CK0, no microorganism inoculated; CK1, only *Ggt* inoculated; Z1, inoculated with *Ggt* and Z-14 when seeds were sown; Z2, treated the same as Z1 and each pot was irrigated with 20 ml of Z-14 broth on the 7th day after sowing.

As Figure 3A shows, multivariate vectors were transformed into uncorrelated principal component (PC) vectors for soil samples collected at 9 days post-sowing after inoculation for 216 h, with PC1 and PC2 explaining 26.522 and 19.641% of the total variance. The separation among the four treatments was evident. In Figure 3B is the PCA for soil samples at 19 days post-sowing after inoculation for 216 h, in which PC1 and PC2, respectively, account for 24.181 and 15.072% of the total variance, respectively. Compared with the 9th-day samples, the position of the CK0 treatment was unchanged whereas CK1 moved toward the positive half axis of PC1 and PC2 and mostly coincided with CK0; meanwhile, Z1 migrated slightly toward the negative half axis of PC1, and Z2 migrated toward the negative half axis of PC2 and now partially overlapped with Z1. At day 9 since seeds were sowed, the four treatments were positioned differently in the PCA quadrants and separated from each other, indicating that inoculation with pathogen and Z-14 significantly impacted the functional diversity of soil microorganisms. However, by the 19th day, CK0 and CK1 now almost overlapped, indicating the influence of pathogens upon microbial functional diversity had gradually disappeared. Meanwhile, Z1 and Z2 approached each other and partially overlapped, indicating the influence of addition times of Z-14 was gradually weakened. The main components of carbon source metabolism were significantly changed by the Z-14 inoculation, and the carbon source utilization capacity was significantly stronger in Z2 than in the other treatments. Duplicate inoculation of Z-14 improved the utilization capacity for carbohydrates, amino acids, and carboxylic acids by wheat rhizospheric soil microorganisms (Supplementary Table S3). The functional diversity of rhizospheric microorganisms reveals differences in the ecological functioning of soil microorganisms, which profoundly impacts soil formation, material cycling, and fertility changes (Su et al., 2020). In recent work, applying microbial agents improved the functional diversity of soil microorganisms, enhanced their community-level ecological functions, and rendered the soil ecosystem more stable (Chakraborty et al., 2019). Li et al. (2021) reported that combining microbes enhanced microbial utilization and changed soil microbial functional structure in the rhizosphere of ryegrass. Here, after the Z-14 inoculation, the soil microbial community made better use of carbon sources, thus favoring beneficial microorganisms and improving microbial diversity.

### Effects of *Bacillus subtilis* Z-14 on wheat rhizospheric bacterial structure and diversity

Using the Illumina Miseq PE300 platform for sequencing, 1,195,153 effective bacterial sequences with an average length of 418 bp were obtained from the 24 soil samples (Supplementary Figures S2, S3). The Shannon index on the 9th day was greatest under CK1 but least under the Z2 treatment. The difference was significant between the two treatments. This suggested adding the fungus *Ggt* increased the soil bacterial community evenness yet strain Z-14 quickly decreased, but the influence of both *Ggt* and Z-14 upon the Shannon index was eliminated over time. These results are consistent with Wan et al. (2017), who found that *B. amyloliquefaciens* SN16-1 treatments featured the lowest values for Shannon and Simpson diversity indices. The *F. oxysporum* f. sp. *lycopersici* treatment showed the highest values, though all groups had similar values at 40 days (Supplementary Table S4).

The OTUs—excluding those unclassified—were organized into nine bacterial taxonomic groups at the phylum level, these accounting for 94.42 ~ 95.40% of total bacteria across all samples, with the Actinobacteria, Proteobacteria, Acidobacteria, Chloroflexi, and Firmicutes being the top-5 phyla, all of which were found in each soil sample (Supplementary Figure S4; Supplementary Table S5). Similarly, Proteobacteria, Actinobacteria, Chloroflexi, and Acidobacteria were the dominant phyla in a tea plantation area of Sichuan Agricultural University in China, but Proteobacteria was the most dominant phylum (Wang et al., 2019), in contrast to our study. The application of strain Z-14 decreased the relative abundance of Actinobacteria and Chloroflexi, but increased that of Firmicutes; the *Ggt* fungus did not significantly influence the bacterial relative abundances.

**Figure 4:**
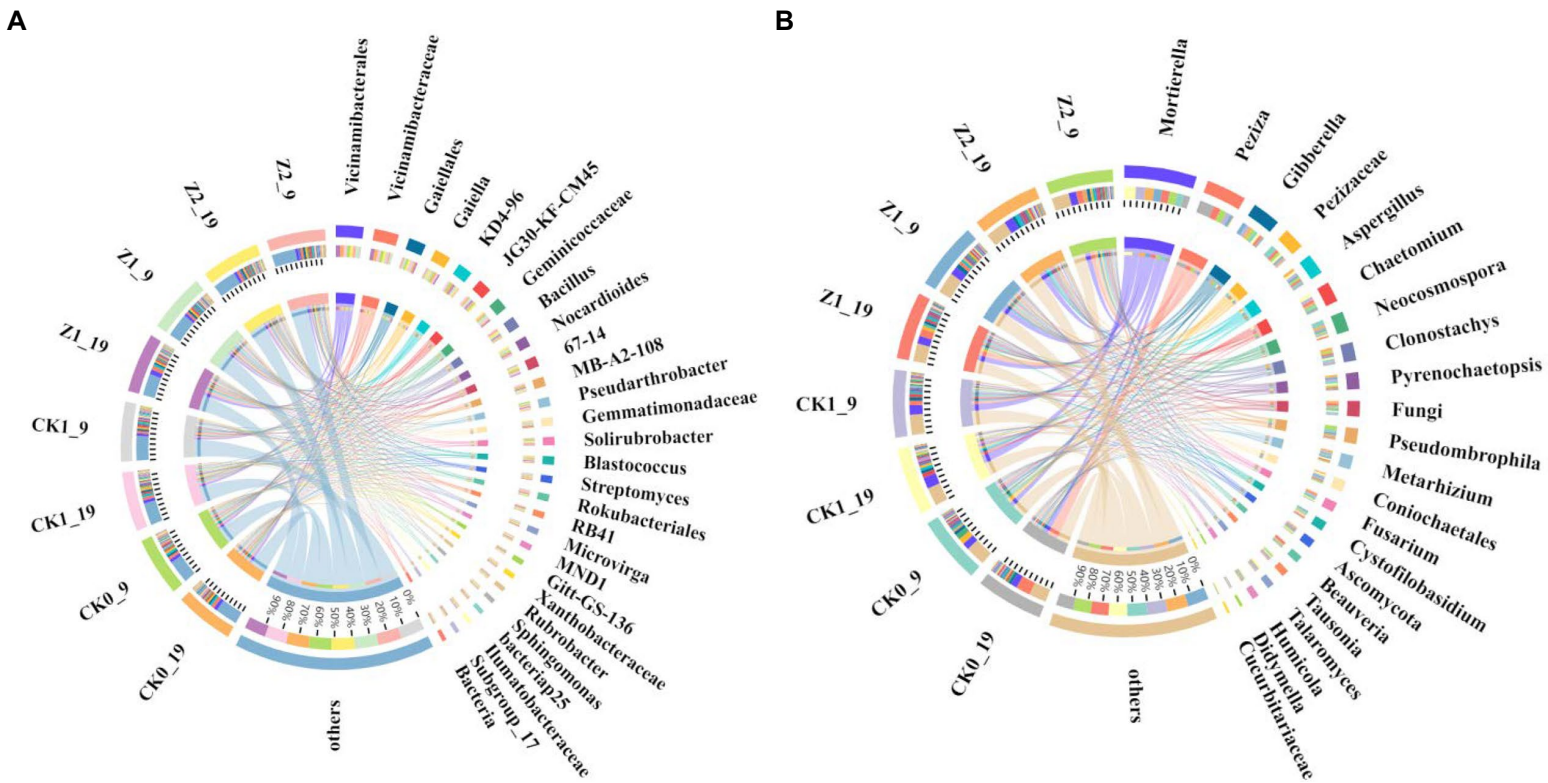
Relative abundances of dominant bacterial **(A)** and fungal **(B)** genera from the wheat rhizosphere sampled on the days 9 and 19 after sowing. CK0, no microorganism inoculated; CK1, only *Ggt* inoculated; Z1, inoculated with *Ggt* and Z-14 when seeds were sown; Z2, treated the same as Z1 and each pot was irrigated with 20 ml of Z-14 broth on the 7th day after sowing.

At the genus level, comparing the relative abundances of the top-20 classified genera revealed that six displayed significant differences among the treatments (Figure 4A; Supplementary Figure S5). *Ggt* significantly increased the relative abundance of *Streptomyces*, while Z-14 decreased that of KD4-96, MB-A2-108, and *Pseudarthrobacter*. Hence, *Ggt* stimulated the proliferation of some bacteria while Z-14 inhibited some. Applying strain Z-14 significantly improved the relative abundance of *Bacillus* in soil, while *Ggt* did not significantly affect it. The relative abundance of *Bacillus* differed significantly between treatments Z1 and Z2, indicating the extra addition of Z-14 promotes the abundance of *Bacillus* in soil. Meanwhile, between sampling times, Z1 was negligibly changed (2.73% → 2.67%) yet the difference for Z2 was significant (4.74% → 3.79%); this suggests Z-14 can effectively colonize the wheat rhizosphere, but when the quantity of viable bacteria exceeded a certain range, the number of Z-14 gradually decreased over time (Supplementary Table S6). On the contrary, the relative abundance of *Streptomyces* in roots after *S. fulvissimus* FU14-coated wheat seeds were sown was unchanged 4 weeks later (Ricardo et al., 2020). Summarizing our research and previous findings, the amount of biocontrol bacteria in soil declines and disappears over time. Yet, continuous addition increases the number of biocontrol bacteria and prolongs their existence in the plant rhizosphere, thus increasing the control effect and generating an extensive and profound impact there (Cuppels et al., 2013).

**Figure 5:**
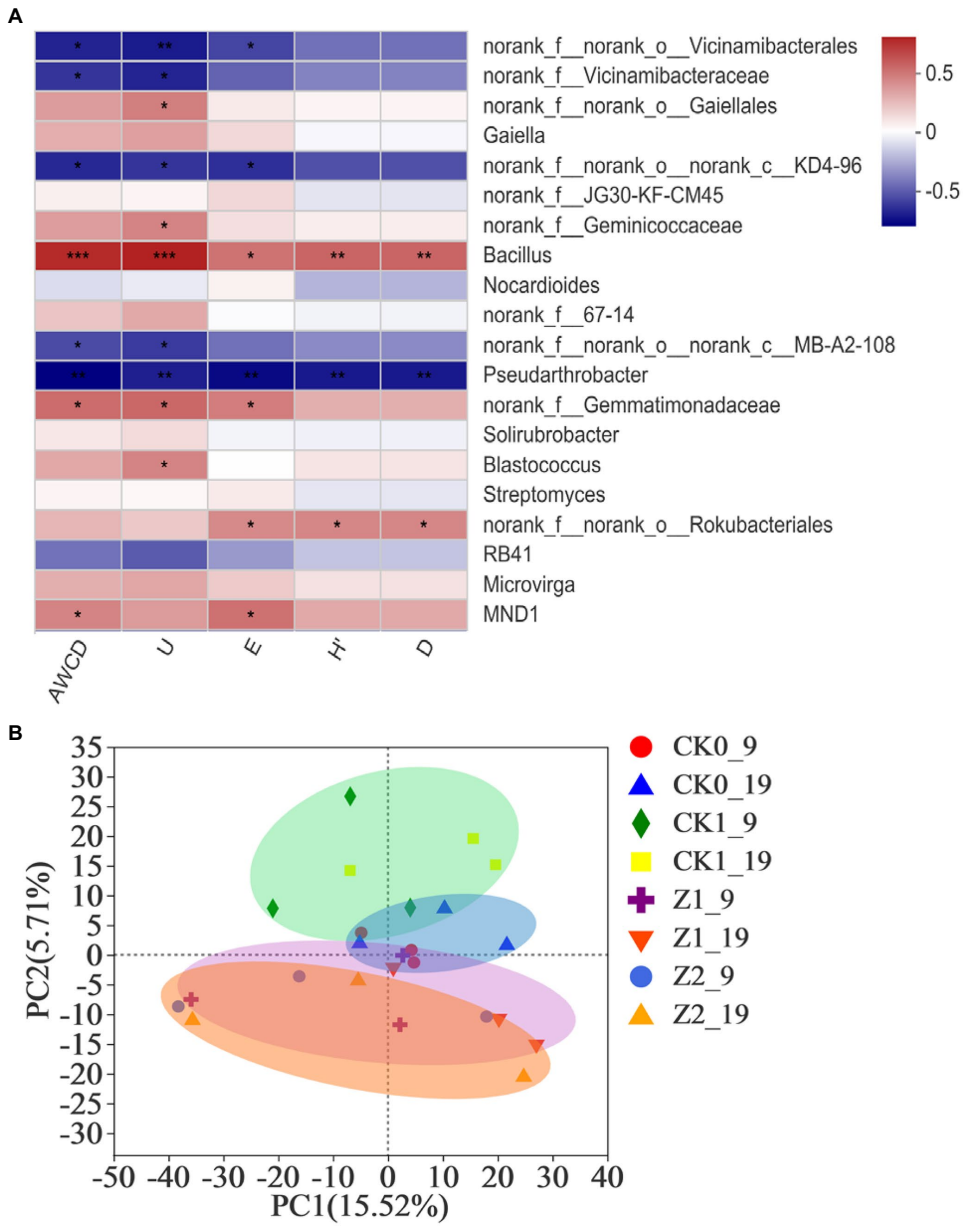
Heatmap showing the relationships between microbial functional diversity indexes and relative abundances of bacterial community structure (at the genus level) in wheat rhizospheric soil treated with the fungus pathogen *Ggt* and/or strain Z-14, based on Spearman’s rank correlation **(A)**, and principal component analysis of the soil bacterial community from wheat rhizosphere sampled on the 9th and 19th day after sowing **(B)**. The color bar indicates the values of correlation coefficients. ^*^p < 0.05, ^**^p < 0.01, ^***^p < 0.001. CK0, no microorganism inoculated; CK1, only *Ggt* inoculated; Z1, inoculated with *Ggt* and Z-14 when seeds were sown; Z2, treated the same as Z1 and each pot was irrigated with 20 ml of Z-14 broth on the 7th day after sowing. Circle coloreds green, blue, purple, and orange represent treatments CK0, CK1, Z1, and Z2, respectively.

The Spearman correlation heatmap for the relative abundances of top-20 classified genera from rhizospheric soil vis-à-vis the microbial functional diversity indexes appears in Figure 5A. *Bacillus* showed significant positive correlations with AWCD (*p* < 0.001), U (*p* < 0.001), H′ (*p* < 0.01), D (*p* < 0.01), and E (*p* < 0.05), while the abundances of Gaiellales, Geminicoccaceae, Gemmatimonadaceae, *Blastococcus*, Rokubacteriales, and *MND1* showed significant positive correlations with some of these diversity indexes. Conversely, abundances of Vicinamibacteralse, Vicinamibacteraceae, KD4-96, MB-A2-108, and *Pseudarthrobacter* had significant negative correlations with some indexes. Our comprehensive analysis shows that *Bacillus* played a dominant role in affecting the microbial functional diversity of the wheat rhizosphere. Chen et al. (2017) demonstrated that the community composition of bacteria determined their metabolic activity and functional diversity in soils treated with different irrigation salinities. Furthermore, Pearson’s correlations revealed significant correlations between soil bacterial metabolic activity and community composition during tea planting (Wang et al., 2019), findings consistent with ours here.

PCA revealed no significant difference among the treatments in the direction of PC1 axis; but, along the PC2 axis both CK1 and Z2 were positioned the furthest, implying differences between them were the most significant (Figure 5B). Treatments Z1 and Z2 mostly overlapped, implying little difference between them; hence, adding more of strain Z-14 seemed to not change bacterial diversity. The *Ggt* treatment samples were mainly positioned the positive half axis of PC2, while the strain Z-14 treatment samples lay on its negative half axis, indicating opposite effects of pathogen and antagonist on this second principal component. No-microbe added treatment samples clustered about the origin of PCA quadrant system, and different sampling times (9th vs. 19th day) had no influence on PC1 or PC2. In the previous study (Wan et al., 2017), soil treated with the biocontrol agent *B. amyloliquefaciens* SN16-1 or plant pathogen *F. oxysporum* differed significantly from the control in the PCA, a result in line with our findings above.

**Figure 6:**
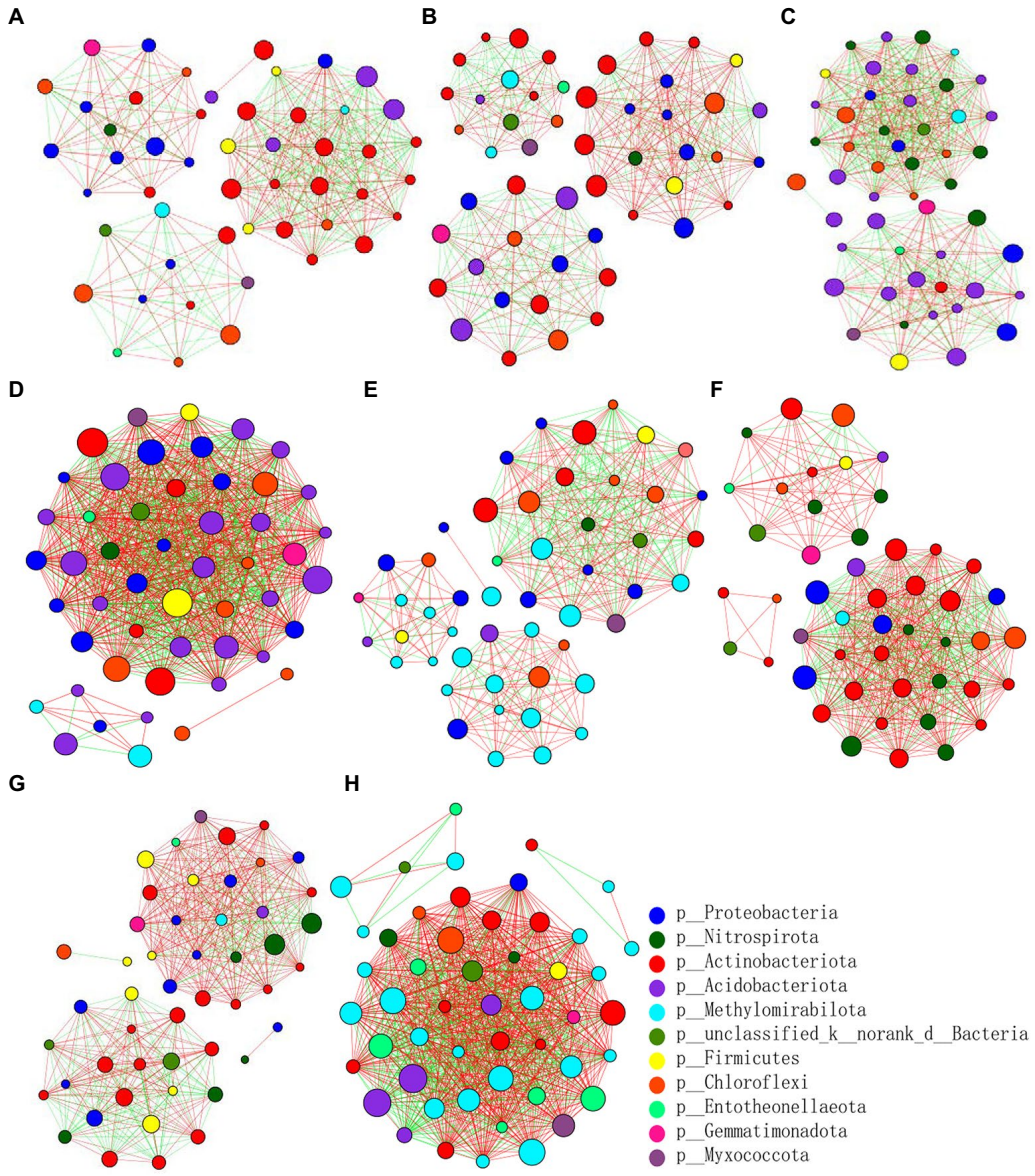
Pearson’s correlation network analyses of bacterial communities from the wheat rhizospheric soil sampled on the 9th **(A–D)** and 19th day **(E–H)** after sowing. CK0, no microorganism inoculated; CK1, only *Ggt* inoculated; Z1, inoculated with *Ggt* and Z-14 when seeds were sown; Z2, treated the same as Z1 and each pot was irrigated with 20 ml of Z-14 broth on the 7th day after sowing. **(A,E)** represent CK0; **(B,F)** represent CK1; **(C,G)** represent Z1; **(D,H)** represent Z2. Nodes represent genera, with their size conveying relative abundance. Lines between nodes denote correlations between the nodes they connect; red and green colors indicate positive and negative correlations, respectively.

At the genus level, the differences and interactions of bacterial communities in the four treatments were analyzed by co-occurrence network analyses, which convey the main topological properties of microbial community correlations (Figure 6). On the 9th day since sowing, treatment Z2 had the most positive links, followed by Z1. However, the number of links increased by the 19th day post-sowing: that of Z2 was still greatest, but the number in CK1 now exceeded that in Z1. Furthermore, treatment Z2 had the largest positive linkages and relative connections, demonstrating that multiple inoculations of the Z-14 biocontrol agent significantly enhanced the positive number of links between bacterial communities (Table 2). Similarly, the relative abundance of B1408 was positively correlated with many plant-growth promoting rhizospheric bacteria, yet significantly negatively correlated with disease index (Han et al., 2019). Other work found cultured root and soil microbial communities with more nodes and links demonstrated higher disease suppression and their frequency of positive links rose in tandem with inoculation of functional microbiota (Liu et al., 2020).

**Table 2:** Pearson’s correlation network analyses of bacterial communities from wheat rhizospheric soil sampled on days 9 and 19 after sowing.

Network index	CK0_9	CK1_9	Z1_9	Z2_9	CK0_19	CK1_19	Z1_19	Z2_19
Total links	400	401	569	877	400	549	492	874
Positive links	217	247	292	552	238	369	341	594
Average degree	16.00	16.04	22.76	35.08	16.32	22.88	20.08	34.96
Relative connections	8.00	8.02	11.38	17.54	8.16	11.44	10.04	17.48

CK0, no microorganism inoculated; CK1, only Ggt inoculated; Z1, inoculated with Ggt and Z-14 when wheat was planted; Z2, treated the same as Z1 and each pot was irrigated with 20 ml of Z-14 broth on the 7th day after sowing.

### Effects of *Bacillus subtilis* Z-14 on wheat rhizospheric fungal structure and diversity

Using the Illumina MiSeq PE300 sequencing platform, we obtained 1,693,100 valid ITS sequences with an average length of 245 bp from the 24 soil samples (Supplementary Figures S6, S7). The application of pathogens and antagonists reduced the ACE and Chao1 indexes of samples collected on the 9th day post-sowing, but no significant difference was observed among the four treatments on the 19th day, suggesting the effects of pathogens and antagonists on both indexes waned over time (Supplementary Table S7). Inoculation with both microbes increased the Shannon index on the 19th day, this was affected more significantly by the antagonist Z-14, while the Simpson index was significantly lower than the control; this result demonstrated a relatively long-term impact of both microbes on these two indexes. Similarly, the fungus FOC and its biocontrol agent B1408 decreased the ACE and Chao1 indexes of fungal community diversity in comparison with the control rhizosphere (Han et al., 2019).

The OTUs—except those unclassified—were organized into seven fungal taxonomic groups at the phylum level, accounting for 95.08% ~ 96.94% of total fungi across all samples, with Ascomycota constituting their majority of 68.75% ~ 78.30%, followed by Mortierellomycota and Basidiomycota in all samples (Supplementary Figures S7, S8; Supplementary Table S8). Ros et al. (2017) reported Ascomycota were the most abundant phylum, accounting for 71.39% of the total fungal OTUs obtained from substrates using a mixture of compost and peat, followed by Basidiomycota and Zygomycota. These findings imply a similar fungal structure of plant rhizospheres irrespective of region, vegetative cover, substrate variety, and so forth, indicating high innate stability of rhizospheric fungal communities (Ricardo et al., 2020).

At the genus level, the dominant *Mortierella* occurred in all soil samples, and its relative abundance was increased by both fungus *Ggt* and strain Z-14 on day 19 after seeds were sown. Meanwhile, Z-14 increased the relative abundance of *Gibberella*, *Aspergillus*, Ascomycota, and *Clonostachys*. Nevertheless, *Ggt* increased the relative abundance of *Chaetomium*, but this effect was weakened by Z-14 (Figure 4B; Supplementary Figure S9). Conversely, the prevalence of *Pseudombrophila* was boosted by Z-14 but inhibited by *Ggt* (Supplementary Table S9). *Mortierella* is an underestimated natural resource that produces two cyclic peptides originating from bacteria (Jacob et al., 2021). *Gibberella* secrete secondary metabolite gibberellins, a critical plant hormone and involved in many plant processes, such as growth and development (Marcela et al., 2020). *Aspergillus* has the unique ability to decompose complex organics and could be applied to foster crop nutrition and growth (Velayuthan et al., 2021). Yet, the genus *Chaetomium* is considered pathogenic and causes many plant diseases (Alam et al., 2021). Inoculation of Z-14 effectively increased the relative abundance of beneficial genera and decreased that of pathogenic genera in soil, thereby augmenting its biocontrol effect.

**Figure 7:**
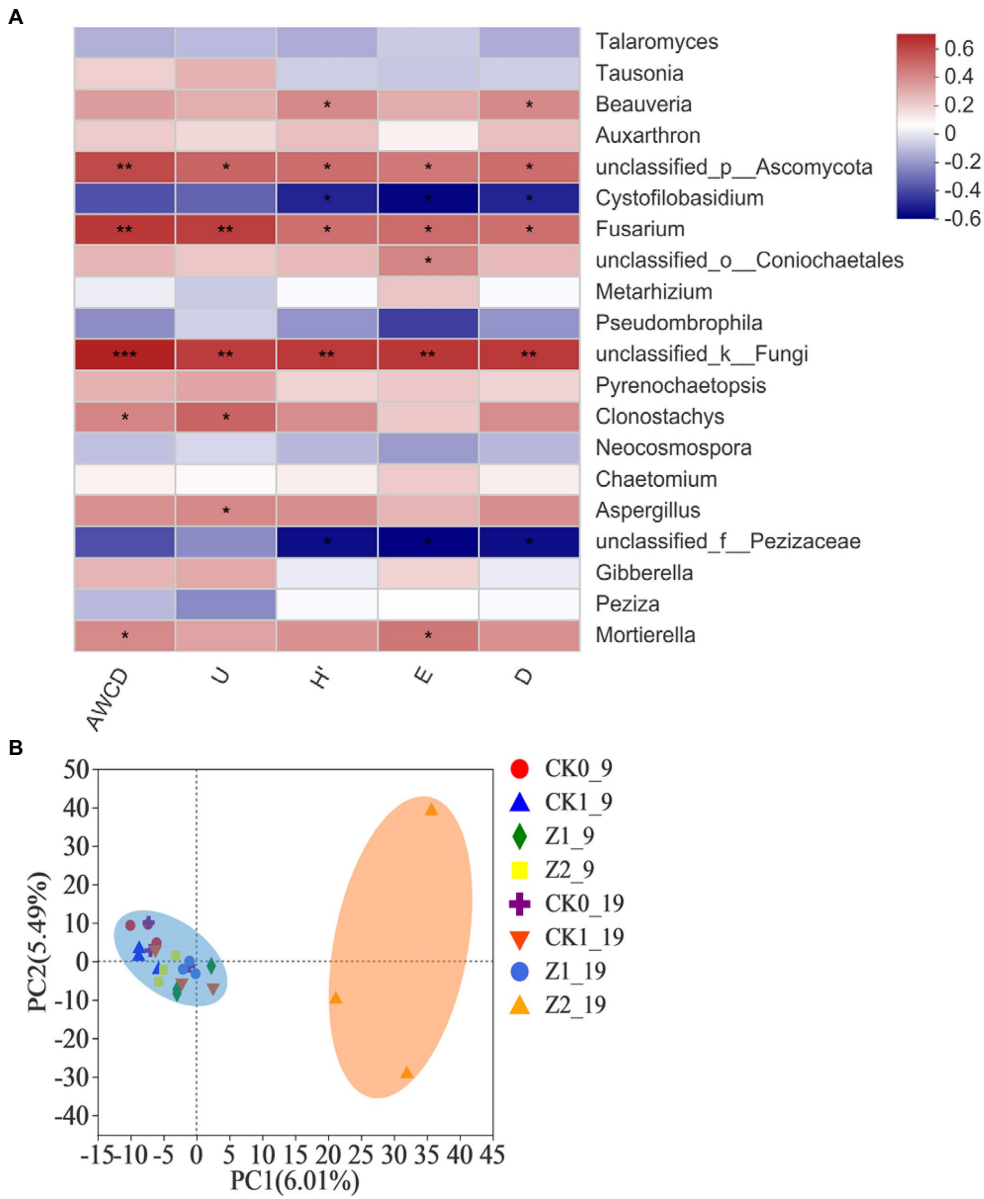
Heatmap showing the relationships between microbial functional diversity indexes and relative abundances of fungal community structure (at the genus level) in wheat rhizospheric soil treated with the fungus pathogen *Ggt* and biocontrol agent Z-14, based on Spearman’s rank correlation **(A)**, and principal component analysis of the fungal community from wheat rhizospheric soil sampled on 9th day and 19th day after sowing **(B)**. The color bar indicates the values of correlation coefficients. ^*^p < 0.05, ^**^p < 0.01, ^***^p < 0.001. CK0, no microorganism inoculated; CK1, only *Ggt* inoculated; Z1, inoculated with *Ggt* and Z-14 when seeds were sown; Z2, treated the same as Z1 and each pot was irrigated with 20 ml of Z-14 broth on the 7th day after sowing.

The Spearman correlation heatmap for the relative abundances of the top-20 classified fungal genera vis-à-vis the microbial functional diversity indexes is presented in Figure 7A. The relative abundance of unclassified fungi was positively correlated with AWCD (*p* < 0.001) as well as U, H′, D, and E (*p* < 0.01). Likewise, the abundances of *Fusarium* and unclassified Ascomycota had significant positive correlations with all five diversity indexes. A correlation analysis between functional diversity indexes and fungal structures of the wheat rhizosphere has not been reported before. Our comprehensive analysis shows that fungal genera positively associated with functional diversity indexes are mostly those genera whose relative abundance increased in response to strain Z-14 inoculation, suggesting this antagonist figures prominently in changing soil functional diversity and fungal abundances.

PCA showed that the Z2 samples collected on 19th day post-sowing, which gathered along the positive axis of PC1, were separated from the others aggregating together on its negative axis, but no significant separation was detected among treatments on the PC2 axis (Figure 7B). These results demonstrated that the addition of phytopathogenic *Ggt* did not influence fungal diversity of the wheat rhizosphere but increased culture time and Z-14 did so significantly. The latter’s influence depended on the multiple application of strain Z-14 to ensure a relatively high dose of it in soil and a relative long actuation duration. Both a low dosage of Z-14 (Z1) and short actuation duration (Z2_9) of the antagonist in the wheat rhizosphere did not significantly influence the fungal diversity. The survival of biocontrol inoculants in rhizosphere can promote plant health in the early phase and then stimulate plant systemic-acquired resistance to extend this effect against future disease pressure (Kang et al., 2019). In fact, although the fungus *Ggt* and bacterial strain Z-14 evidently affected the physiology of wheat seedlings, neither caused severe fungal community shifts in the wheat rhizosphere. Their effects may be attenuated by high microbial diversity that makes crop system more resilient and predictable (Ricardo et al., 2020).

**Figure 8:**
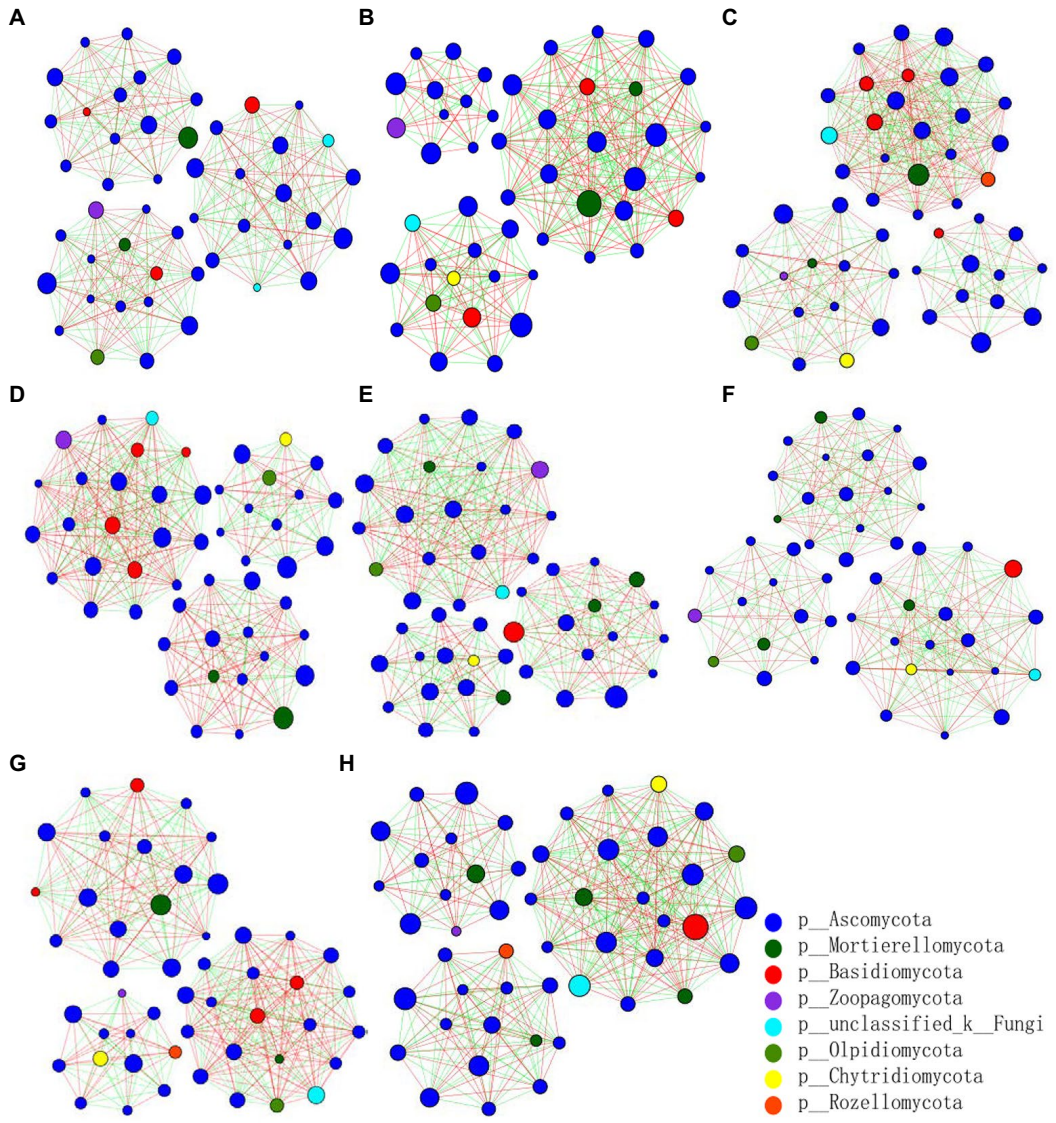
Pearson’s correlation network analyses of fungal communities from rhizospheric soil sampled on the 9th **(A–D)** and 19th day **(E–H)** after sowing. CK0, no microorganism inoculated; CK1, only *Ggt* inoculated; Z1, inoculated with *Ggt* and Z-14 when seeds were sown; Z2, treated the same as Z1 and each pot was irrigated with 20 ml of Z-14 broth on the 7th day after sowing. **(A,E)** represent CK0; **(B,F)** represent CK1; **(C,G)** represent Z1; **(D,H)** represent Z2. Nodes represent genera, with their size conveying relative abundance. Lines between nodes denote correlations between the nodes they connect; red and green colors indicate positive and negative correlations, respectively.

The fungal abundance correlation networks were built to analyze the differences and interactions of fungal communities in the four treatments on the 9th and 19th day post-sowing (Figure 8). On the 9th day, the number of links was significantly higher in Z1 and Z2 than in the CK0 and CK1 treatments, being least under CK0. On the 19th day, Z2 had the most links, followed by Z1, with CK1 having the fewest. The positive linking numbers and relative connections of treatments Z1 and Z2 were greater than those of CK0 or CK1. The number of links among members within a fungal community increased soon after the *Ggt* application, but decreased significantly later on, while strain Z-14 significantly increased the fungal community interactions (Table 3). Pathogen invasion significantly disrupted microbial associations when compared with the healthy tomato whose microbial associations were more complex (Zhang et al., 2021). In our study, more positive interactions were found with the inoculation of strain Z-14, indicating that synergies among microbes were enhanced.

**Table 3:** Pearson’s correlation network analyses of fungal communities from wheat rhizospheric soil sampled on days 9 and 19 after sowing.

Network index	CK0_9	CK1_9	Z1_9	Z2_9	CK0_19	CK1_19	Z1_19	Z2_19
Total links	361	392	424	408	386	385	392	414
Positive links	177	189	212	239	192	183	236	201
Average degree	15.04	15.68	16.96	16.32	15.76	15.71	16.00	16.25
Relative connections	7.52	7.84	8.48	8.16	8.00	7.86	8.00	8.28

CK0, no microorganism inoculated; CK1, only Ggt inoculated; Z1, inoculated with Ggt and Z-14 when wheat was planted; Z2, treated the same as Z1 and each pot was irrigated with 20 ml of Z-14 broth on the 7th day after sowing.

### Genome sequencing and functional genomic analysis of *Bacillus subtilis* Z-14

The functional genes related to the colonization ability of strain Z-14 belonged to four categories: motility and chemotaxis, biofilm formation, polysaccharide degradation, and plant growth promotion. In the Z-14 genome are 53 genes related to motility and chemotaxis, 40 genes for biofilm synthesis, 7 genes promoting plant growth, and 13 genes for polysaccharide degradation (Supplementary Figure S10; Supplementary Table S10).

The predicted secondary metabolite gene clusters of strain Z-14 from anti-SMASH are shown in Supplementary Table S11. Ten secondary metabolite gene clusters exist in the strain, nine types of which are substances with antimicrobial activity. The similarity between cluster 1 and the rhizocticin synthetic gene cluster derived from bgc0000926_C1 was only 22%. In addition, two unknown functional genes, Cluster 6 (Type III PKS) and Cluster 9 (NRP), were also found. These gene clusters may encode secondary metabolites with new structures, which merit isolation and identification.

Chen et al. (2007) analyzed the genes relevant to *Bacillus* colonization and proved that *epsA-O* and *γ-PGA* operons controlling the extracellular polysaccharide component of biofilm contain genes related to chemotaxis and flagellum assembly. The plant-growth-promoting activity of *B. subtilis* arises in part from its production of plant growth regulators, such as 2, 3-butanediol and 3-indoleacetic acid. Genes encoding these products were also found in strain Z-14, which further emphasized the basis of its biocontrol ability.

The genome of Z-14 also contains abundant secondary metabolic gene clusters, another known significant feature of *B. subtilis* as a biocontrol agent. These clusters mainly encode nonribosomal peptide synthetase (NRPS) and polyketide synthases (PKS; Chen et al., 2007). Compounds synthesized by the NRPS or PKS synthesis pathway usually have antifungal or antibacterial activities. Lipopeptides synthesized by the NRPS pathway can also induce resistance in plants, and mediate the formation of biocontrol biofilm and rhizospheric colonization (Ongena and Jacques, 2008). We found 10 NRPS and PKS synthase gene clusters in Z-14’s genome, these encoding nine antimicrobial substances and two unknown secondary metabolites. The existence of these secondary metabolic gene clusters is extremely important for conferring the broad-spectrum antimicrobial ability to this strain, which provides a basis better understanding effective disease prevention mechanism of Z-14.

## Conclusion

Application of *B. subtilis* Z-14 controls wheat take-all disease with a treatment efficacy of 66.25%, and significantly increases the carbon source utilization and functional diversity indexes of soil microorganisms. A second inoculation of Z-14 significantly increases the abundance of *Bacillus* in soil, confirming the colonization ability of Z-14 strain in the wheat rhizosphere, and enhances the positive links between bacterial communities. Strain Z-14 influences the fungal diversity. However, this influence depends on its repeated application, so that soil receives a relatively high dose of biocontrol agent in the soil, and relative long actuation duration. More positive interactions arise after inoculation with Z-14, indicating that synergies among microbes are enhanced. Furthermore, 113 functional genes related to colonization ability and 10 secondary metabolite gene clusters exist in this strain. This research provides a timely basis for comprehensively understanding the effective disease prevention mechanism of strain Z-14.

## Data availability statement

The datasets presented in this study can be found in online repositories. The names of the repository/repositories and accession number(s) can be found at: https://www.ncbi.nlm.nih.gov/, PRJNA813967 and PRJNA805073.

## Author contributions

DZ and DW contributed to the study conception and design. DZ, ZL, and XZ wrote the manuscript. ZL, JX, XZ, and TG performed the experiments. ZL, JX, and SD performed the statistical analyses. All authors contributed to the article and approved the submitted version.

## Funding

This work was supported by the Hebei Provincial Natural Science Foundation of China (No. C2019204210), the Hebei Provincial Key Research and Development Project of China (Nos. 20326509D and 20322907D), and the Local Science and Technology Development Fund Projects of Hebei Province Guided by the Central Government (No. 206Z6502G).

## Conflict of interest

The authors declare that the research was conducted in the absence of any commercial or financial relationships that could be construed as a potential conflict of interest.

## Publisher’s note

All claims expressed in this article are solely those of the authors and do not necessarily represent those of their affiliated organizations, or those of the publisher, the editors and the reviewers. Any product that may be evaluated in this article, or claim that may be made by its manufacturer, is not guaranteed or endorsed by the publisher.

## Footnotes

^1^ https://www.ncbi.nlm.nih.gov/sra/PRJNA805073

^2^ https://www.ncbi.nlm.nih.gov/sra/PRJNA813967

^3^ www.majorbio.com

^4^ http://circos.ca/
