# Supplementary material for: Influence of Bacillus subtilis strain Z-14 on microbial communities of wheat rhizospheric soil infested with Gaeumannomyces graminis var. tritici
Source: Front Microbiol. 2022 Sep 2;13:923242. doi: 10.3389/fmicb.2022.923242 (PMC9479631; doi:10.3389/fmicb.2022.923242)
Supplement: Supplementary file 1 [file Table_1.DOCX]

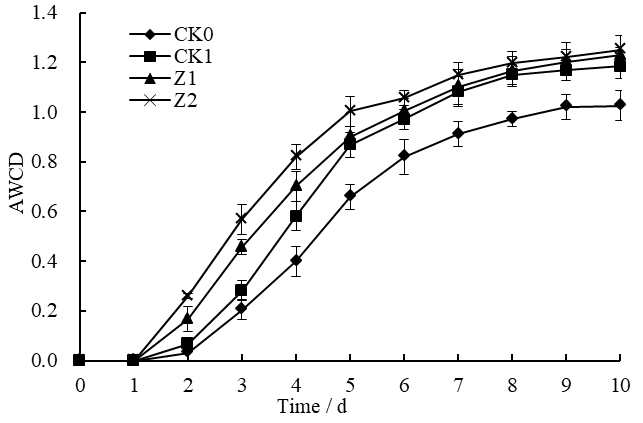

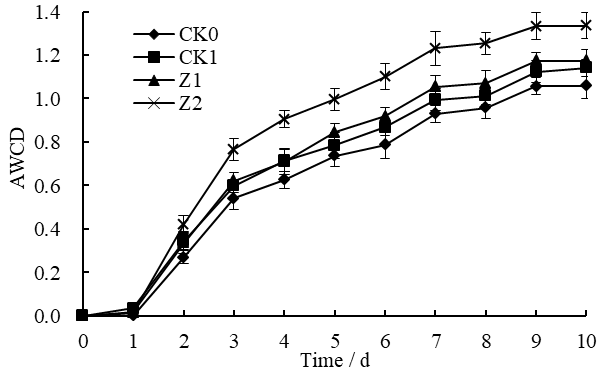


B

A

**Figure S1** Average well-color development (AWCD) of Biolog Eco-microplates during the incubation of microbial communities from the wheat rhizosphere sampled on the 9th day (A) and 19th day (B) after sowing.


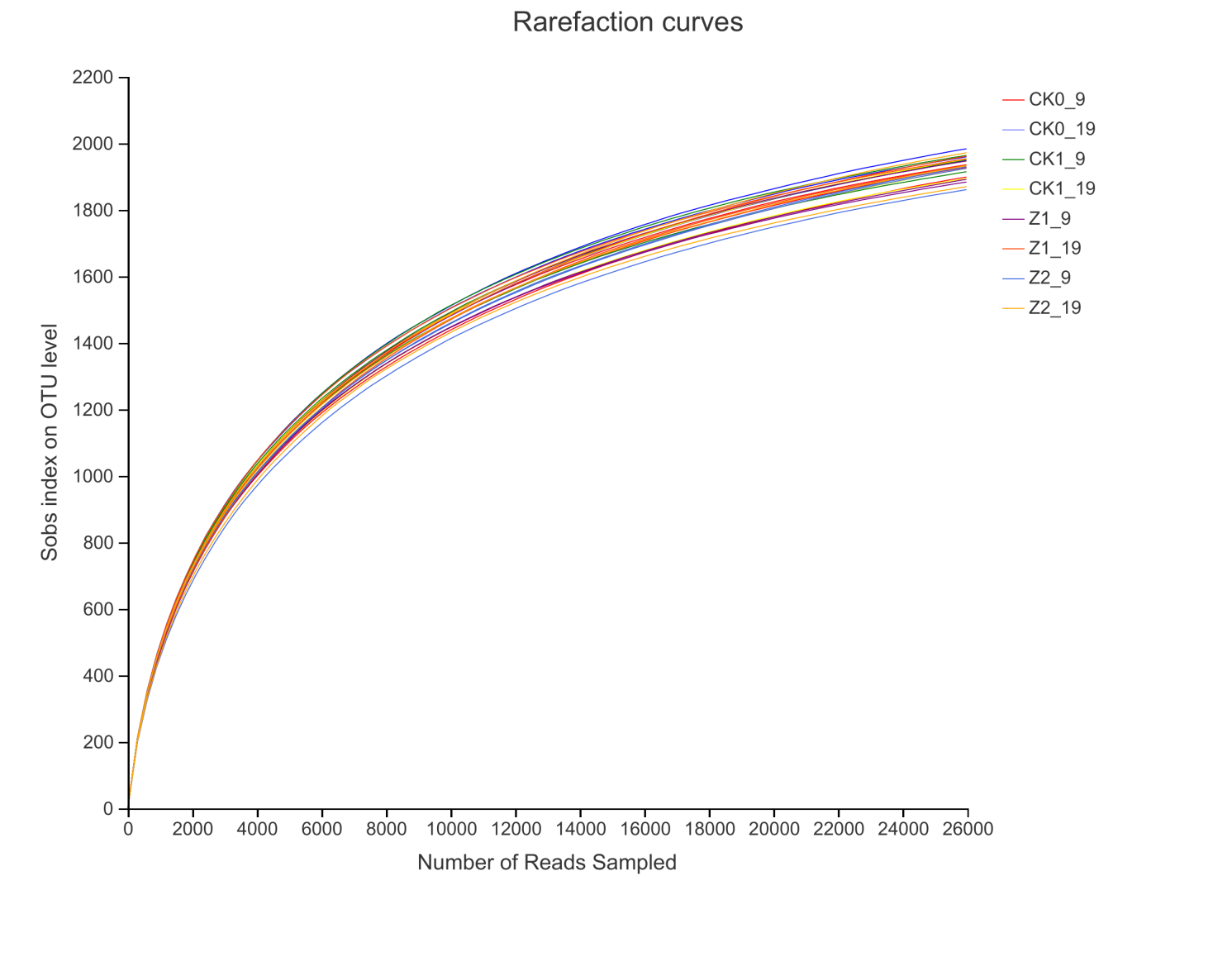


**Figure S2** Rarefaction curves of bacteria in wheat rhizospheric soil sampled on the 9th and 19th day after planting. CK0, no microorganism was inoculated; CK1, only *Ggt* was inoculated; Z1, inoculated with *Ggt* and Z-14 when wheat was sown; Z2, treated the same as Z1 and each pot was then irrigated with 20 mL of Z-14 broth on the 7^th^ day after sowing. The same below.


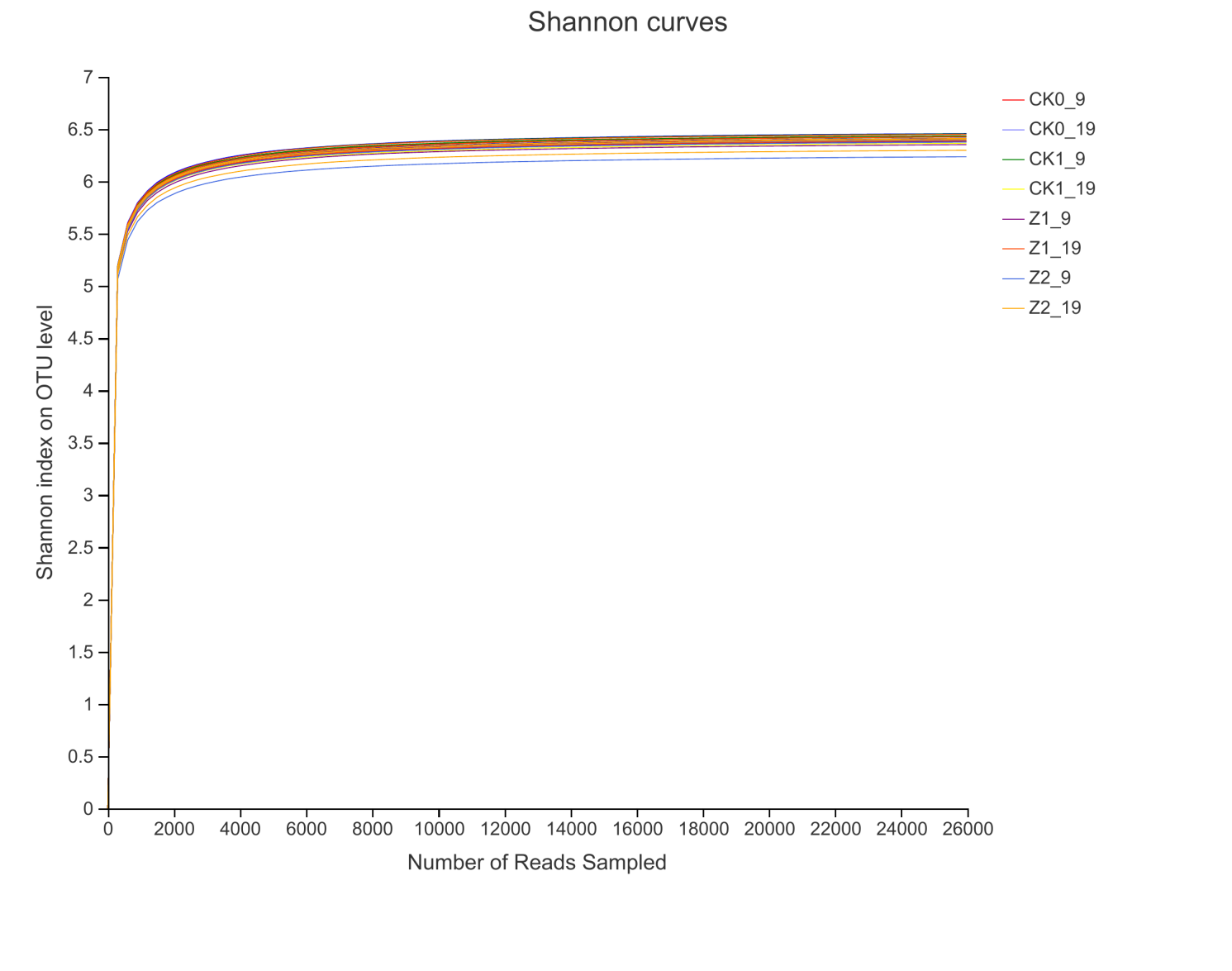


**Figure S3** Shannon curves of bacteria in wheat rhizospheric soil sampled on the 9th and 19th day after sowing.


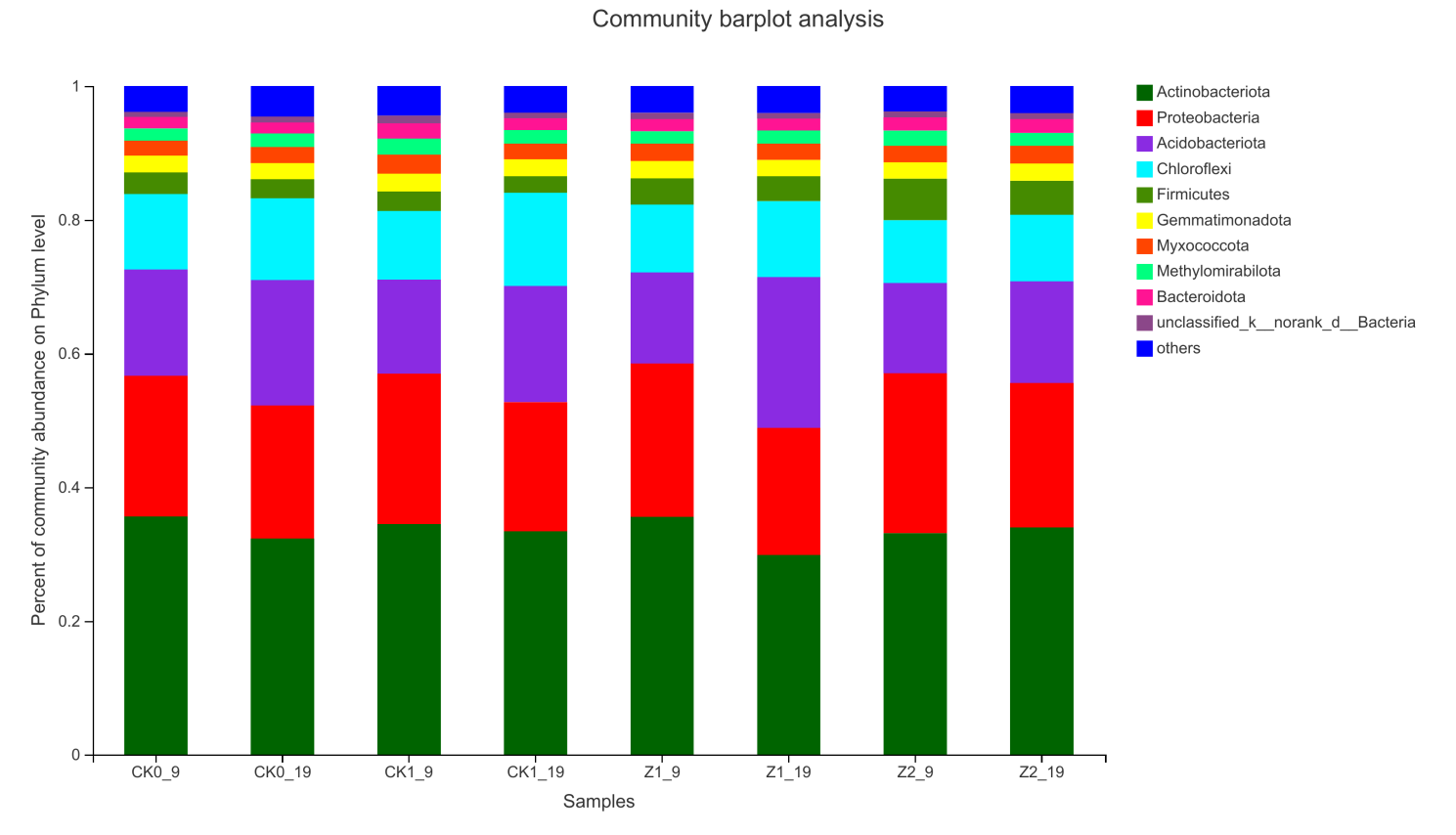


**Figure S4** Relative abundance of bacterial community structure in wheat rhizospheric soil sampled on the 9th and 19th day after sowing (at the phylum level).

**
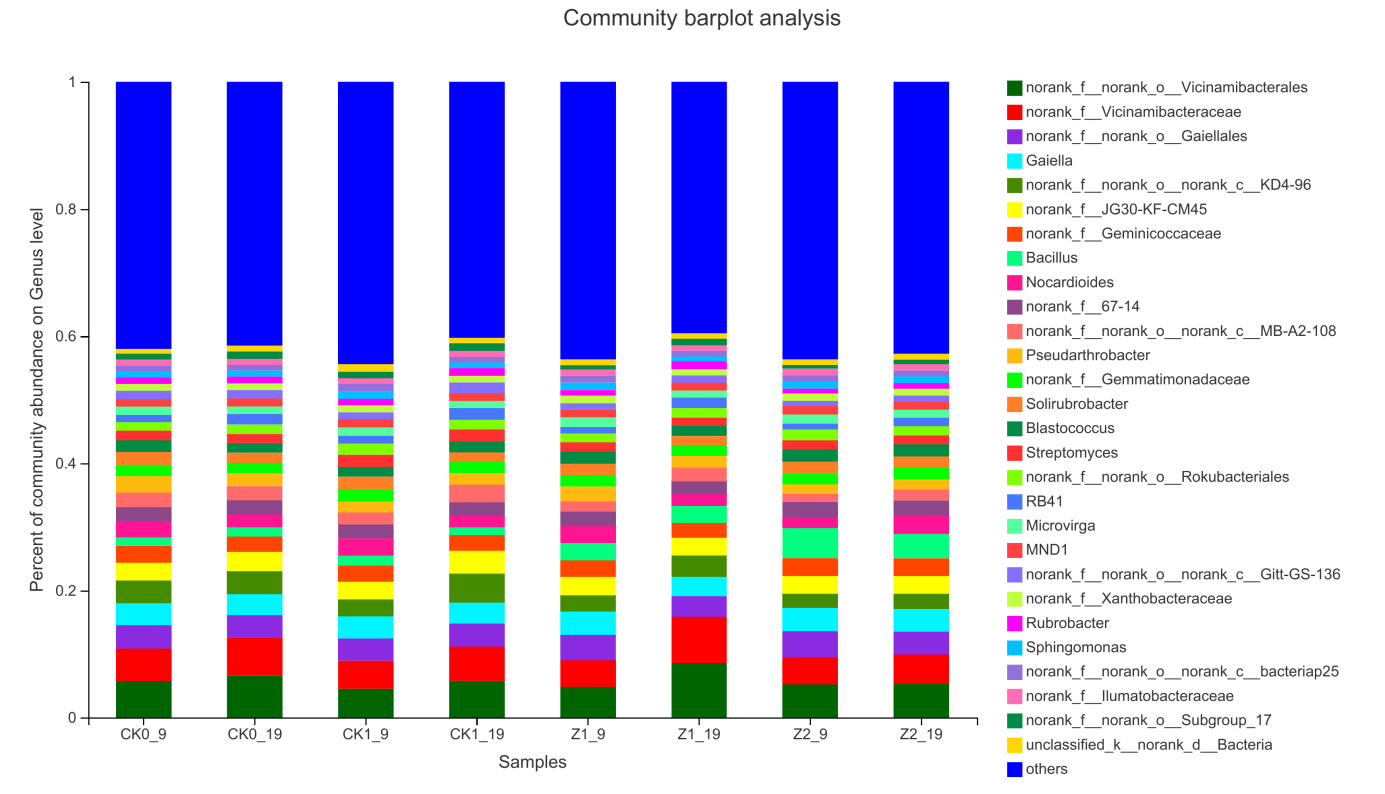
**

**Figure S5** Relative abundance of bacterial community structure in wheat rhizospheric soil sampled on the 9th and 19th day after sowing (at the genus level).


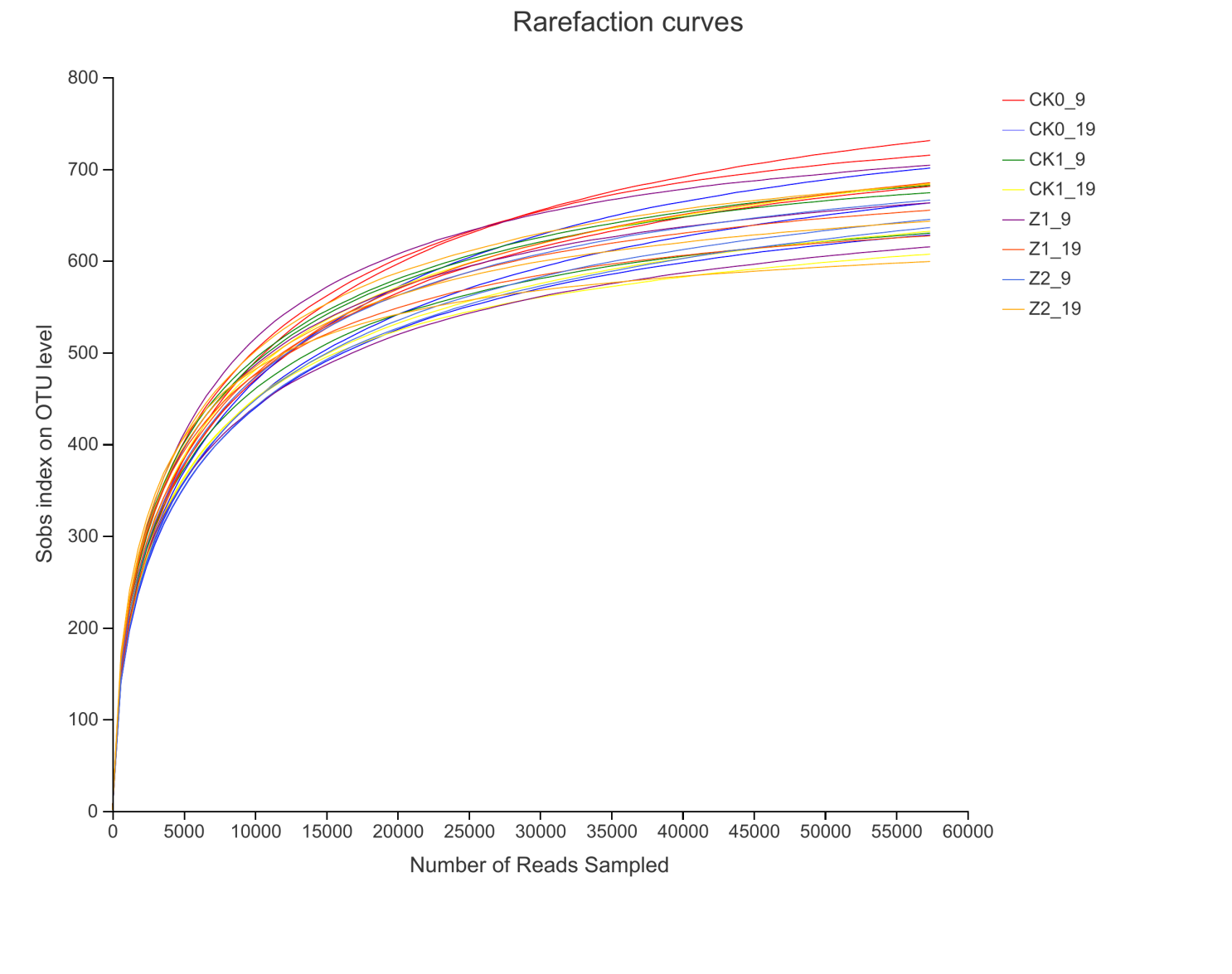


**Figure S6** Rarefaction curves of fungi in wheat rhizospheric soil sampled on the 9th and 19th day after sowing.


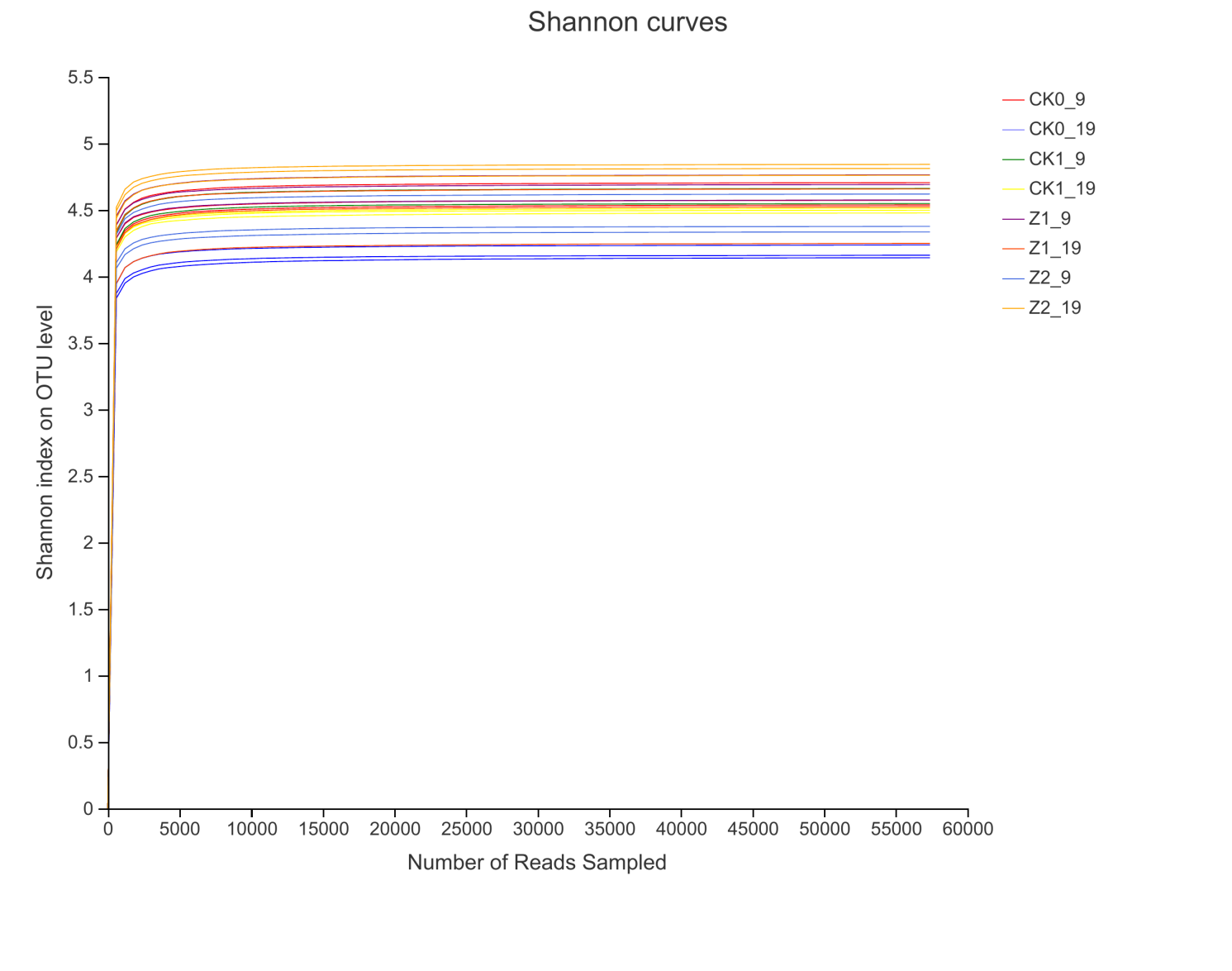


**Figure S7** Shannon curves of fungi in wheat rhizospheric soil sampled on the 9th and 19th day after sowing.


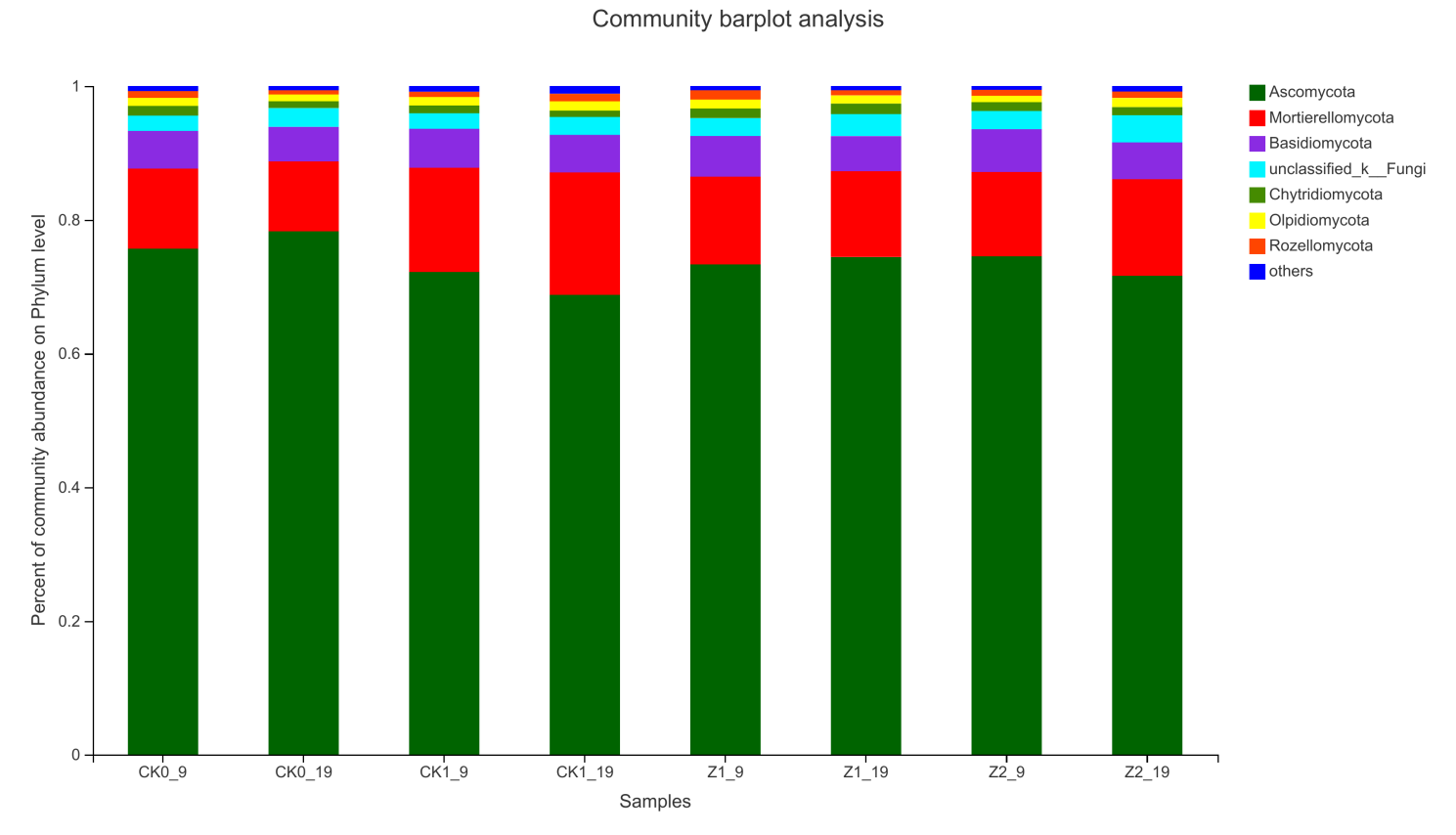


**Figure S8** Relative abundance of fungal community structure in wheat rhizospheric soil sampled on the 9th and 19th day after sowing (at the phylum level).


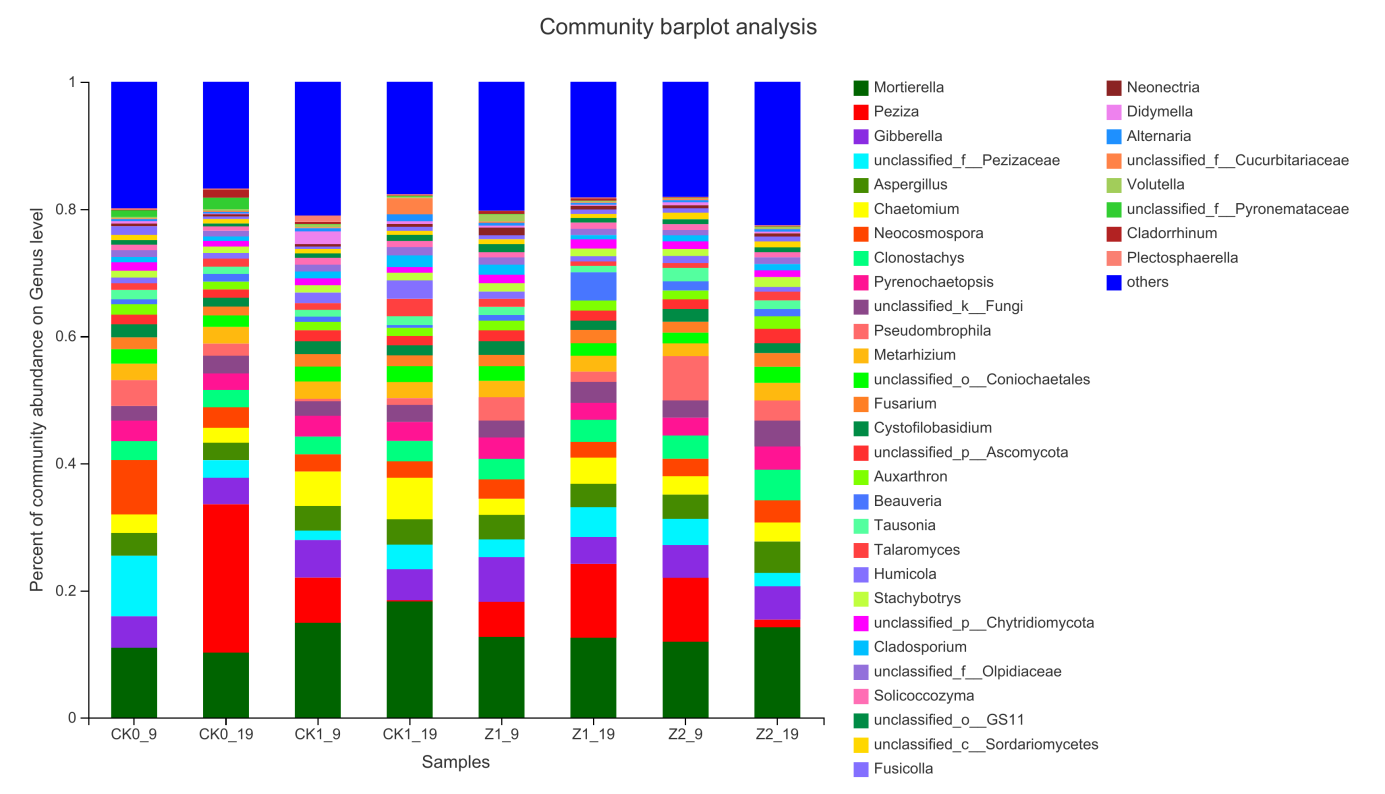


**Figure S9** Relative abundance of fungal community structure in wheat rhizospheric soil sampled on the 9th and 19th day after sowing (at the genus level).


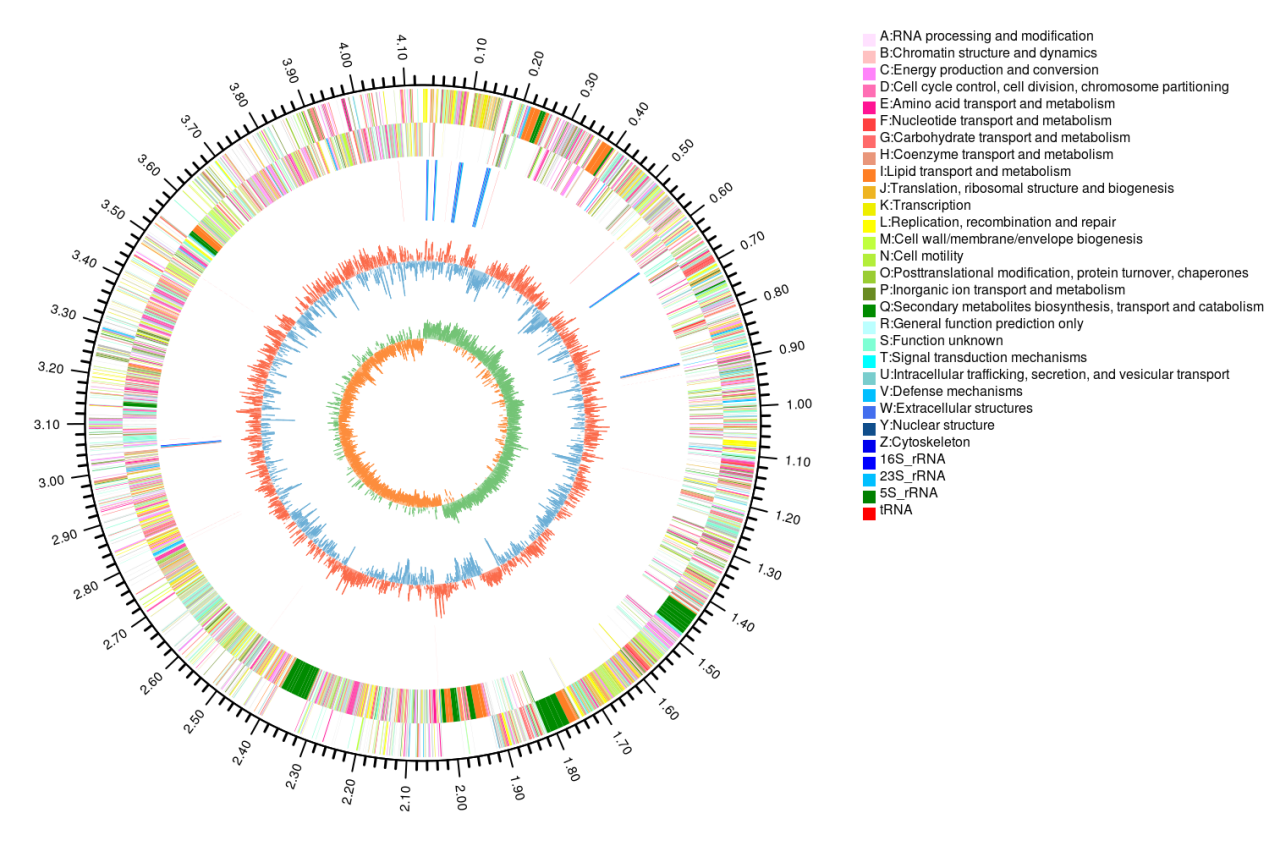


**Figure S10** Circular genomic map of *Bacillus* *subtilis* strain Z-14. From the outer to the inner circle are scale marks of the genome (Mb); genes on the forward strand, genes on the reverse strand; rRNA and tRNA; the GC ratio and GC skew. Genes are color-coded according to their COG (Clusters of Orthologous Group) category.

**Table S1** Distribution of six types of carbon sources in the Biolog Eco-Plate.

| Chemical guild | Plate number | Substrate | Chemical formula | 9th day | | 19th day | |
| --- | --- | --- | --- | --- | --- | --- | --- |
|  |  |  |  | PC1 | PC2 | PC1 | PC2 |
| Miscellaneous | B1 | Pyruvic acid methyl ester | C_4_H_6_O_3_ | 0.573 | 0.275 | 0.415 | −0.390 |
|  | G2 | Glucose-1-phosphate | C_6_H_13_O_9_P | 0.809 | 0.326 | 0.399 | 0.400 |
|  | H2 | D,L-α-Glycerol phosphate | C_3_H_9_O_6_P | 0.771 | 0.331 | 0.734 | 0.333 |
| Polymers | C1 | Tween 40 | − | −0.260 | −0.443 | 0.216 | −0.355 |
|  | D1 | Tween 80 | − | −0.554 | −0.300 | −0.284 | −0.432 |
|  | E1 | α-Cyclodextrin | C_36_H_60_O_30_ | −0.229 | 0.162 | 0.141 | 0.380 |
|  | F1 | Glycogen | (C_6_H_10_O_5_)n | 0.043 | 0.521 | 0.763 | 0.162 |
| Carbohydrates | G1 | D-Cellobiose | C_12_H_12_O_11_ | −0.153 | −0.623 | 0.504 | 0.468 |
|  | H1 | α-D-Lactose | C_12_H_12_O_11_ | −0.700 | −0.283 | 0.166 | 0.455 |
|  | A2 | Methyl-D-glucoside | C_7_H_14_O_6_ | 0.941 | −0.104 | 0.702 | −0.068 |
|  | B2 | D-Xylose | C_5_H_10_O_5_ | 0.657 | 0.061 | 0.607 | 0.416 |
|  | C2 | i-Erythritol | C_4_H_10_O_4_ | 0.146 | −0.577 | 0.327 | 0.722 |
|  | D2 | D-Mannitol | C_6_H_14_O_6_ | 0.715 | −0.265 | 0.019 | 0.690 |
|  | E2 | *N*-Acetyl-D-glucosamine | C_8_H_15_NO_6_ | 0.908 | 0.001 | −0.007 | −0.057 |
| Carboxylic acids | F2 | D-Glucosaminic acid | C_6_H_13_NO_6_ | 0.013 | −0.314 | −0.210 | 0.025 |
|  | A3 | D-Galactonic acid latone | C_6_H_10_O_6_ | 0.785 | −0.191 | 0.395 | 0.420 |
|  | B3 | D-Galacturonic acid | C_6_H_10_O_7_ | 0.472 | −0.040 | 0.502 | 0.299 |
|  | C3 | 2-Hydroxy benzoic acid | C_7_H_6_O_3_ | 0.424 | −0.308 | −0.113 | 0.486 |
|  | D3 | 4-Hydroxy benzoic acid | C_7_H_6_O_3_ | −0.247 | 0.150 | −0.559 | −0.177 |
|  | E3 | γ-Hydroxy butyric acid | C_4_H_8_O_3_ | −0.216 | 0.814 | 0.623 | −0.183 |
|  | F3 | Itaconic acid | C_5_H_6_O_4_ | 0.200 | 0.791 | 0.632 | −0.144 |
|  | G3 | α-Keto butyric acid | C_4_H_6_O_3_ | −0.619 | 0.540 | 0.717 | −0.174 |
|  | H3 | D-Malic acid | C_4_H_6_O_5_ | 0.204 | −0.627 | 0.505 | −0.073 |
| Amino acids | A4 | L-Arginine | C_4_H_14_N_4_O_2_ | 0.022 | 0.173 | 0.064 | −0.297 |
|  | B4 | L-Asparagine | C_4_H_8_N_2_O_3_ | 0.302 | 0.276 | 0.587 | −0.019 |
|  | C4 | L-Phenylalanine | C_9_H_11_NO_2_ | 0.147 | 0.691 | 0.727 | 0.049 |
|  | D4 | L-Serine | C_3_H_7_NO_3_ | 0.649 | 0.193 | 0.527 | −0.461 |
|  | E4 | L-Threonine | C_4_H_9_NO_3_ | 0.281 | 0.649 | 0.626 | −0.243 |
|  | F4 | Glycyl-L-glutamic acid | C_7_H_12_N_2_O_5_ | −0.385 | 0.739 | 0.511 | −0.668 |
| Amines/amides | G4 | Phenylethylamine | C_8_H_11_N | −0.451 | 0.714 | 0.308 | −0.754 |
|  | H4 | Putrescine | C_4_H_12_N_2_ | 0.684 | 0.035 | 0.638 | −0.402 |

**Table S2** Primers used for the fungal ITS1 region and bacterial 16S rDNA V3~V4 region.

| Gene | Primer | Primer sequence (5' to 3') |
| --- | --- | --- |
| Fungus ITS1 | ITS1F | CTTGGTCATTTAGAGGAAGTAA |
|  | ITS2 | GCTGCGTTCTTCATCGATGC |
| Bacteria 16S rDNA V3~V4 | 338F | ACTCCTACGGGAGGCAGCA |
|  | 806R | GGACTACHVGGGTWTCTAAT |

**Table S3** Metabolic utilization on different carbon sources by microbial communities from wheat rhizospheric soil sampled on the 9th and 19th day after sowing.

| Carbon source | 9th day | | 19th day | |
| --- | --- | --- | --- | --- |
|  | PC1 | PC2 | PC1 | PC2 |
| Carbohydrates | 5 | 2 | 3 | 5 |
| Amino acids | 1 | 3 | 5 | 2 |
| Carboxylic acids | 4 | 4 | 6 | 2 |
| Polymers | 1 | 2 | 1 | 1 |
| Amines/amides | 2 | 1 | 1 | 2 |
| Miscellaneous | 3 | 0 | 2 | 1 |

**Table S4** Bacterial community richness and diversity indexes of wheat rhizospheric soil sampled on the 9th and 19th day after sowing.

| Time | Tr | OUT | ACE | Chao1 | Simpson | Shannon | Coverage |
| --- | --- | --- | --- | --- | --- | --- | --- |
| 9 d | CK0 | 1921.00 | 2239.68±25.56a | 2209.86±39.80a | 0.00±0.00a | 6.40±0.03ab | 98.38% |
|  | CK1 | 1943.33 | 2229.71±25.56a | 2223.62±45.81a | 0.00±0.00a | 6.44±0.01a | 98.43% |
|  | Z1 | 1907.67 | 2193.34±31.28a | 2178.60±23.53a | 0.00±0.00a | 6.38±0.03ab | 98.44% |
|  | Z2 | 1905.33 | 2236.00±63.30a | 2203.26±62.74a | 0.00±0.00a | 6.34±0.09b | 98.34% |
| 19 d | CK0 | 1964.33 | 2275.42±32.84a | 2249.46±36.29a | 0.00±0.00a | 6.44±0.02a | 98.37% |
|  | CK1 | 1924.00 | 2245.42±54.32a | 2221.40±73.78a | 0.00±0.00a | 6.39±0.02a | 98.36% |
|  | Z1 | 1950.33 | 2254.98±38.39a | 2218.60±26.376a | 0.00±0.00a | 6.42±0.03a | 98.39% |
|  | Z2 | 1925.00 | 2238.07±84,65a | 2223.11±70.41a | 0.00±0.00a | 6.37±0.06a | 98.36% |

*Note*: Tr, treatments; CK0, no microorganism inoculated; CK1, only *Ggt* inoculated; Z1, inoculated with *Ggt* and Z-14 when wheat was planted; Z2, treated the same as Z1 and each pot was irrigated with 20 mL of Z-14 broth on the 7th day after planting. Different lowercase letters in the same column indicate significant differences between treatments at the 5% level (*P* < 0.05).

**Table S5** Relative abundance of bacterial community structure in wheat rhizospheric soil sampled on the 9th and 19th day after sowing (at the phylum level).

| Phylum | CK0_9 (%) | CK1_9 (%) | Z1_9 (%) | Z2_9 (%) | CK0_19 (%) | CK1_19 (%) | Z1_19 (%) | Z2_19 (%) |
| --- | --- | --- | --- | --- | --- | --- | --- | --- |
| Actinobacteria | 35.62±1.19a | 34.48±0.45a | 35.55±0.55a | 33.13±3.03ab | 32.32±2.20ab | 33.40±1.34ab | 29.88±2.41b | 34.00±4.62ab |
| Proteobacteria | 21.05±0.65a | 22.51±1.87a | 22.97±4.44a | 23.90±5.25a | 19.90±2.15a | 19.32±2.23a | 18.98±1.45a | 23.90±5.25a |
| Acidobacteria | 15.88±2.11a | 14.06±2.81a | 13.59±5.21a | 13.52±4.31a | 18.76±4.31a | 17.41±4.14a | 22.60±5.77a | 15.21±9.28a |
| Chloroflexi | 11.30±0.30bc | 10.28±0.70bc | 10.17±2.37bc | 9.40±1.76c | 12.25±1.24ab | 13.92±0.68a | 11.35±0.55bc | 9.95±1.88bc |
| Firmicutes | 3.24±0.17cde | 2.90±0.37cde | 3.92±0.80c | 6.21±1.07a | 2.81±0.47de | 2.45±0.09e | 3.73±0.53cd | 5.09±0.17b |
| Gemmatimonadota | 2.50±0.06a | 2.66±0.31a | 2.56±0.41a | 2.44±0.38a | 2.45±0.34a | 2.52±0.24a | 2.44±0.32a | 2.57±0.46a |
| Myxococcota | 2.24±0.06a | 2.86±0.41a | 2.65±0.55a | 2.46±0.48a | 2.39±0.38a | 2.35±0.37a | 2.42±0.30a | 2.65±0.50a |
| Methylomirabilota | 1.81±0.13a | 2.38±0.50a | 1.83±0.01a | 2.27±0.86a | 2.01±0.13a | 2.07±0.20a | 1.99±0.13a | 1.93±0.12a |
| Bacteroidota | 1.75±0.33a | 2.28±0.47a | 1.81±0.44a | 1.99±0.14a | 1.66±0.13a | 1.75±0.41a | 1.76±0.18a | 2.06±0.60a |

*Note*: CK0, no microorganism inoculated; CK1, only *Ggt* inoculated; Z1, inoculated with *Ggt* and Z-14 when wheat was planted; Z2, treated the same as Z1 and each pot was irrigated with 20 mL of Z-14 broth on the 7th day after planting. The 9 and 19 denote the set of soil samples respectively taken on the 9th and 19th day after planting. Values shown are the mean of three replicates ± SD (standard deviation). Different lowercase letters within the same row indicate significant differences between treatments at the 5% level (*P* < 0.05). The same below.

**Table S6** Relative abundance of bacterial community structure in wheat rhizospheric soil sampled on the 9th and 19th day after sowing (at the genus level).

| Genus | CK0_9 (%) | CK1_9 (%) | Z1_9 (%) | Z2_9 (%) | CK0_19 (%) | CK1_19 (%) | Z1_19 (%) | Z2_19 (%) |
| --- | --- | --- | --- | --- | --- | --- | --- | --- |
| Vicinamibacterales | 5.72±0.98a | 4.53±1.18a | 4.86±2.24a | 5.21±4.04a | 6.57±1.51a | 5.76±1.41a | 8.56±2.75a | 5.27±3.94a |
| Vicinamibacteraceae | 5.14±0.73a | 4.39±0.89a | 4.15±1.75a | 4.22±3.16a | 6.04±1.64a | 5.44±1.59a | 7.32±2.06a | 4.64±3.27a |
| Gaiellales | 3.67±0.19a | 3.52±0.17a | 4.00±0.58a | 4.16±0.88a | 3.50±0.35a | 3.58±0.37a | 3.21±0.28a | 3.63±0.60a |
| *Gaiella* | 3.43±0.01a | 3.48±0.31a | 3.64±0.55a | 3.68±0.86a | 3.33±0.31a | 3.29±0.21a | 3.03±0.25a | 3.50±0.67a |
| KD4-96 | 3.62±0.16ab | 2.62±0.57b | 2.58±1.39b | 2.19±1.22b | 3.57±0.78ab | 4.58±1.46a | 3.36±0.26ab | 2.46±1.17b |
| JG30-KF-CM45 | 2.79±0.13a | 2.84±0.18a | 2.91±0.10a | 2.79±0.21a | 3.04±0.14a | 3.59±1.43a | 2.81±0.15a | 2.75±0.07a |
| Geminicoccaceae | 2.65±0.04a | 2.51±0.18a | 2.62±0.16a | 2.82±0.86a | 2.42±0.25a | 2.46±0.26a | 2.31±0.18a | 2.83±0.83a |
| *Bacillus* | 1.32±0.18d | 1.57±0.19d | 2.73±0.55c | 4.74±0.80a | 1.48±0.29d | 1.24±0.06d | 2.67±0.37c | 3.79±0.30b |
| *Nocardioides* | 2.48±0.19a | 2.73±1.20a | 2.60±0.33a | 1.67±0.39a | 1.99±0.10a | 1.81±0.07a | 1.84±0.27a | 2.96±1.43a |
| 67-14 | 2.29±0.04a | 2.17±0.13a | 2.32±0.26a | 2.45±0.39a | 2.22±0.41a | 2.13±0.05a | 2.02±0.23a | 2.32±0.53a |
| MB-A2-108 | 2.24±0.10ab | 1.90±0.45ab | 1.57±0.74ab | 1.27±0.81b | 2.26±0.32ab | 2.74±0.82a | 2.16±0.25ab | 1.75±0.88ab |
| *Pseudarthrobacter* | 2.64±0.33a | 1.71±0.36b | 2.36±0.94ab | 1.47±0.57b | 1.99±0.06ab | 1.86±0.19ab | 1.84±0.14ab | 1.54±0.49b |
| Gemmatimonadaceae | 1.72±0.08a | 1.84±0.29a | 1.83±0.34a | 1.77±0.39a | 1.66±0.26a | 1.74±0.18a | 1.69±0.21a | 1.85±0.38a |
| *Solirubrobacter* | 2.05±0.03a | 2.07±0.61a | 1.75±0.09a | 1.84±0.40a | 1.61±0.32a | 1.50±0.17a | 1.45±0.14a | 1.80±0.42a |
| *Blastococcus* | 1.85±0.07a | 1.58±0.04a | 1.90±0.16a | 1.90±0.41a | 1.53±0.26a | 1.68±0.22a | 1.59±0.19a | 1.92±0.40a |
| *Streptomyces* | 1.51±0.10b | 1.91±0.18a | 1.49±0.13b | 1.45±0.30b | 1.41±0.15b | 1.90±0.14a | 1.29±0.03b | 1.37±0.28b |
| Rokubacteriales | 1.32±0.10a | 1.74±0.33a | 1.35±0.05a | 1.69±0.63a | 1.50±0.08a | 1.56±0.16a | 1.50±0.06a | 1.43±0.12a |
| RB41 | 1.17±0.07ab | 1.20±0.25ab | 1.05±0.34ab | 0.92±0.54b | 1.66±0.45ab | 1.83±0.46a | 1.66±0.30ab | 1.36±0.69ab |
| *Microvirga* | 1.30±0.11a | 1.34±0.06a | 1.53±0.40a | 1.43±0.39a | 1.17±0.09a | 1.11±0.18a | 1.12±0.09a | 1.26±0.21a |
| MND1 | 1.17±0.02a | 1.35±0.15a | 1.26±0.22a | 1.33±0.35a | 1.25±0.19a | 1.25±0.15a | 1.26±0.10a | 1.27±0.28a |

**Table S7** Fungal community richness and diversity indexes of wheat rhizospheric soil sampled on the 9th and 19th day after sowing.

| Time | Tr | OUT | ACE | Chao1 | Simpson | Shannon | Coverage |
| --- | --- | --- | --- | --- | --- | --- | --- |
| 9 d | CK0 | 709.00 | 755.16±29.06a | 760.53±34.99a | 0.02±0.00a | 4.60±0.09a | 99.85% |
|  | CK1 | 662.00 | 695.38±30.68b | 696.64±25.59b | 0.02±0.00a | 4.62±0.07a | 99.89% |
|  | Z1 | 660.67 | 693.92±38.38b | 693.85±39.60b | 0.02±0.00a | 4.68±0.10a | 99.89% |
|  | Z2 | 649.00 | 697.56±10.47b | 712.38±9.35ab | 0.02±0.00a | 4.64±0.15a | 99.85% |
| 19 d | CK0 | 664.00 | 712.33±42.26a | 709.84±40.62a | 0.06±0.01a | 4.18±0.05c | 99.85% |
|  | CK1 | 641.00 | 683.36±50.16a | 691.76±45.36a | 0.03±0.00b | 4.50±0.02b | 99.87% |
|  | Z1 | 655.67 | 690.66±43.20a | 707.03±58.34a | 0.03±0.02b | 4.48±0.21b | 99.88% |
|  | Z2 | 641.67 | 667.98±50.07a | 678.15±46.00a | 0.02±0.00b | 4.81±0.04a | 99.90% |

**Table S8** Relative abundance of fungal community structure in wheat rhizospheric soil sampled on the 9th and 19th day after sowing (at the phylum level).

| Phylum | CK0_9 (%) | CK1_9 (%) | Z1_9 (%) | Z2_9 (%) | CK0_19 (%) | CK1_19 (%) | Z1_19 (%) | Z2_19 (%) |
| --- | --- | --- | --- | --- | --- | --- | --- | --- |
| Ascomycota | 75.69±1.50ab | 72.16±1.24bc | 73.32±1.16b | 74.56±3.60ab | 78.30±2.55a | 68.75±0.75c | 74.43±3.32ab | 71.61±0.75bc |
| Mortierellomycota | 11.93±0.54e | 15.63±0.70b | 13.11±0.50cd | 12.57±1.12cde | 10.43±2.14de | 18.34±1.65a | 12.83±1.57cd | 14.47±0.84bc |
| Basidiomycota | 5.65±0.27a | 5.84±1.11a | 6.08±0.52a | 6.42±1.43a | 5.16±1.67a | 5.61±0.21a | 5.24±0.80a | 5.49±0.15a |
| Unclassified fungi | 2.31±0.34b | 2.30±0.13b | 2.69±0.30b | 2.73±1.33b | 2.79±0.13b | 2.67±0.56b | 3.29±0.41ab | 4.07±0.38a |
| Chytridiomycota | 1.43±0.93a | 1.17±0.44a | 1.44±0.16a | 1.31±0.34a | 1.05±0.36a | 0.95±0.07a | 1.59±0.57a | 1.22±0.18a |
| Olpidiomycota | 1.21±0.05a | 1.26±0.15a | 1.34±0.44a | 0.99±0.16a | 1.01±0.16a | 1.39±0.72a | 1.19±0.09a | 1.35±0.13a |
| Rozellomycota | 1.03±0.35abc | 0.80±0.18bc | 1.40±0.24a | 0.86±0.05bc | 0.62±0.05c | 1.12±0.37ab | 0.80±0.28bc | 0.94±0.19bc |

*Note*: CK0, no microorganism inoculated; CK1, only *Ggt* inoculated; Z1, inoculated with *Ggt* and Z-14 when wheat was planted; Z2, treated the same as Z1 and each pot was irrigated with 20 mL of Z-14 broth on the day after planting. The 9 and 19 denote the set of soil samples respectively taken on 9th and 19th day after sowing. Values shown are the mean of three replicates ± SD (standard deviation). Different lowercase letters within the same row indicate significant differences between treatments at the 5% level (*P* < 0.05). The same below.

**Table S9** Relative abundance of fungal community structure in wheat rhizospheric soil sampled on the 9th and 19th day after sowing (at the genus level).

| Genus | CK0_9 (%) | CK1_9 (%) | Z1_9 (%) | Z2_9 (%) | CK0_19 (%) | CK1_19 (%) | Z1_19 (%) | Z2_19 (%) |
| --- | --- | --- | --- | --- | --- | --- | --- | --- |
| *Mortierella* | 10.99±0.31de | 14.93±1.00b | 12.70±0.58cd | 11.92±0.62de | 10.24±1.94e | 18.27±1.56a | 12.55±1.46cd | 14.20±0.97bc |
| *Peziza* | 0.00±0.00e | 7.10±1.08bc | 5.53±0.56cd | 10.05±1.57bc | 23.32±3.22a | 0.18±0.08de | 11.63±7.41b | 1.19±0.59de |
| *Gibberella* | 4.95±0.80bc | 5.91±1.18ab | 6.99±0.77a | 5.17±0.32bc | 4.18±0.61c | 4.86±0.78bc | 4.22±0.77c | 5.26±0.42bc |
| Pezizaceae | 9.53±0.61a | 1.47±0.32d | 2.82±0.66bcd | 4.12±0.70bc | 2.74±2.05bcd | 3.85±1.69bc | 4.71±1.13b | 2.10±0.44cd |
| *Aspergillus* | 3.56±0.35bc | 3.88±0.23abc | 3.85±0.71abc | 3.81±0.62abc | 2.73±0.04c | 4.05±0.69ab | 3.64±1.00bc | 4.93±0.77a |
| *Chaetomium* | 2.90±0.73c | 5.41±0.83ab | 2.52±0.71c | 2.89±0.85c | 2.37±0.57c | 6.48±2.32a | 4.15±1.19bc | 3.03±0.25c |
| *Neocosmospora* | 8.59±0.78a | 2.71±0.29bc | 3.04±0.21bc | 2.77±0.51bc | 3.20±0.49bc | 2.61±0.47bc | 2.48±0.54c | 3.48±0.46b |
| *Clonostachys* | 2.96±0.11bcd | 2.80±0.14cd | 3.27±0.29bcd | 3.64±0.87b | 2.74±0.06d | 3.22±0.04bcd | 3.46±0.30bc | 4.78±0.32a |
| *Pyrenochaetopsis* | 3.24±0.52abc | 3.26±0.06abc | 3.33±0.60ab | 2.81±0.19bc | 2.62±0.45c | 2.99±0.26bc | 2.68±0.14bc | 3.66±0.19a |
| Unclassified fungi | 2.31±0.34b | 2.30±0.13b | 2.69±0.30b | 2.73±1.33b | 2.79±0.13b | 2.67±0.56b | 3.29±0.41ab | 4.07±0.38a |
| *Pseudombrophila* | 4.07±3.15ab | 0.34±0.49b | 3.65±2.89ab | 6.95±5.95a | 1.92±2.02ab | 1.07±1.40b | 1.60±2.75ab | 3.17±1.74ab |
| *Metarhizium* | 2.59±0.27ab | 2.75±0.06a | 2.58±0.18ab | 2.00±0.31b | 2.61±0.70ab | 2.52±0.25ab | 2.49±0.22ab | 2.77±0.47a |
| Coniochaetales | 2.28±0.15ab | 2.38±0.16ab | 2.29±0.56abc | 1.69±0.06c | 1.79±0.08bc | 2.51±0.60ab | 1.98±0.20b | 2.53±0.34a |
| *Fusarium* | 1.85±0.24a | 1.95±0.18a | 1.77±0.34a | 1.71±0.30a | 1.38±0.15a | 1.69±0.22a | 2.11±1.16a | 2.16±0.22a |
| *Cystofilobasidium* | 2.04±0.23a | 2.01±0.65a | 2.14±0.78a | 2.01±0.17a | 1.40±0.29a | 1.58±0.32a | 1.42±0.23a | 1.56±0.33a |
| Ascomycota | 1.53±0.33b | 1.74±0.27b | 1.73±0.31b | 1.51±0.24b | 1.32±0.07b | 1.49±0.16b | 1.58±0.25b | 2.23±0.30a |
| *Auxarthron* | 1.64±0.66ab | 1.31±0.07ab | 1.55±0.10ab | 1.38±0.47ab | 1.21±0.30b | 1.27±0.24b | 1.62±0.37ab | 1.98±0.19a |
| *Beauveria* | 0.78±0.23bc | 0.84±0.20bc | 0.88±0.12bc | 1.46±0.27b | 1.19±0.54bc | 0.43±0.13c | 4.46±1.17a | 1.14±0.09bc |
| *Tausonia* | 1.47±0.61a | 1.05±0.25a | 1.27±0.09a | 2.11±1.55a | 1.15±0.26a | 1.42±0.29a | 0.98±0.26a | 1.36±0.19a |
| *Talaromyces* | 1.04±0.08bc | 1.06±0.09bc | 1.27±0.48bc | 0.78±0.13c | 1.30±0.32bc | 2.72±0.48a | 0.74±0.12c | 1.40±0.37b |

**Table S10** Genes related to the colonization ability of *B. subtilis* strain Z-14.

| Colonization function | Functional categories | Types of genes | Number |
| --- | --- | --- | --- |
| Motility- and chemotaxis-related genes | Flagellum structure and assembly | *motB*, *motA*, *fliG*, *fliJ*, *fliL*, *fliM*, *fliY*, *flgB*, *flgC*, *fliE*, *fliF*, *fliK*, *flgD*, *flgE*, *fliZ*, *flip*, *fliQ*, *fliR*, *flhB*, *flhA*, *flhF*, *flhG*, *fliT*, *fliS*, *fliD*, *flgL*, *flgK*, *flgM*, *flgG* | 33 |
|  | Chemotaxis function | *yfmS*, *hemAT*, *mcpC*, *cheV*, *cheB*, *cheW*, *cheC*, *cheD*, *cheR*, *tlpB*, *mcpA*, *tlpA*, *mcpB* | 14 |
|  | Regulating motility and chemotaxis | *flaA*, *cheY*, *cheA*, *fliA*, *swrB* | 6 |
| Biofilm formation-related genes | Biofilm matrix formation | *abrB*, *spoVT*, *kbaA*, *yhgN*, *kind*, *kinA*, *kinC*, *spo0a*, *sinI*, *sinR*, *tasA*, *sipW*, *tapA*, *bslA*, *kinB*, *degP*, *slrR*, *degU*, *degS*, *slrA* | 25 |
|  | Synthesis of extracellular polysaccharides | *epsO*, *epsN*, *epsM*, *epsL*, *epsK*, *epsJ*, *epsI*, *epsH*, *epsG*, *epsF*, *epsE*, *epsD*, *epsC*, *epsB*, *epsA* | 15 |
| Polysaccharide degradation | – | *mall*, *ydhP*, *ysdC*, *celF*, *yhfE*, *xynB*, *xynA*, *xylR*, *xylA*, *xylB*, *bglC*, *bglA*, *bglS* | 13 |
| Plant growth promotion | – | *yhcX*, *dhaS*, *yedL*, *alsD*, *alsR*, *ilvB*, *poxL* | 7 |

**Table S11** Secondary metabolite clusters predicted in the genome of *B. subtilis* strain Z-14.

| Gene cluster | Nucleotide length (bp) | Gene dosage | Cluster type | Genetic similarity ratio (%) | Most similar gene cluster |
| --- | --- | --- | --- | --- | --- |
| Cluster1 | 77609 | 49 | TransATPKS-NRPS | BGC0000926 (22%) | Rhizocticin |
| Cluster2 | 64860 | 43 | NRPS | BGC0000433 (91%) | Surfactin |
| Cluster3 | 86374 | 18 | TransATPKS | BGC0000181 (100%) | Macrolactin H |
| Cluster4 | 108734 | 55 | TransATPKS-NRPS | BGC0001089 (100%) | Bacillaene |
| Cluster5 | 134771 | 65 | TransATPKS-NRPS | BGC0001095 (100%) | Fengycin and Iturin |
| Cluster6 | 41100 | 49 | T3PKS | – | – |
| Cluster7 | 106179 | 60 | TransATPKS | BGC0000176 (100%) | Difficidin |
| Cluster8 | 50509 | 48 | NRPS | BGC0000309 (100%) | Bacillibactin |
| Cluster9 | 68438 | 40 | NRPS | – | – |
| Cluster10 | 41418 | 47 | Other KS | BGC0001184 (100%) | Bacilysin |
